# Genetically modified animals as models of neurodevelopmental conditions: A review of systematic review reporting quality

**DOI:** 10.1177/23982128241287279

**Published:** 2024-10-18

**Authors:** Emma Wilson, Gillian Currie, Malcolm Macleod, Peter Kind, Emily S. Sena

**Affiliations:** 1 Centre for Clinical Brain Sciences, The University of Edinburgh, Edinburgh, UK; 2 Simons Initiative for the Developing Brain, The University of Edinburgh, Edinburgh, UK; 3 Centre for Discovery Brain Sciences, The University of Edinburgh, Edinburgh, UK; 4 Patrick Wild Centre for Autism Research, The University of Edinburgh, Edinburgh, UK

**Keywords:** Evidence synthesis, reporting quality, transparency, neurodevelopmental conditions, epilepsy, autism, intellectual disability, animal models

## Abstract

Using genetically modified animals to model neurodevelopmental conditions helps better our understanding of biology underlying these conditions. Animal research has unique characteristics not shared with clinical research, meaning systematic review methods must be adapted to this context. We aim to evaluate the quantity, characteristics, and reporting quality of systematic reviews which synthesise research using genetically modified animals to model neurodevelopmental conditions. On 23 January 2023, we searched PubMed, Embase, and the Web of Science Core Collection to identify systematic reviews of genetic neurodevelopmental condition animal research where the modified gene was one in a list of 102 genes associated with neurodevelopmental conditions identified through large-scale exome sequencing or *Fmr1*, *Mecp2*, or *Ube3a*. Two independent reviewers screened studies based on full text and assessed the reporting quality of relevant reviews using an adapted version of the PRISMA checklist (PRISMA-Pre). Twelve review publications met our criteria. We found mixed levels of reporting: items such as identifying the publication as a systematic review in the title, search strategies, and funding sources being well reported, and others such as protocol registration and data sharing less well reported. We also identified 19 review registrations via PROSPERO, most of which remain unpublished after their anticipated end dates. Systematic reviews are limited by lack of reporting. Increased awareness of reporting guidelines may help authors increase the transparency and reproducibility, and therefore the reliability, of their systematic reviews.

## Introduction

Up to 40% of neurodevelopmental conditions (NDCs) presenting with intellectual disability (ID), epilepsy, and autism are caused by single genetic alterations, often occurring de novo in that individual (Brunet et al., 2021). These single gene alterations provide an opportunity to develop animal models harbouring those alterations, which may give insights into NDC pathophysiology and treatment more generally, including NDCs with polygenic or environmental causes. Such models can be used to investigate associated cellular, circuit, and behavioural traits. Researchers have developed numerous genetically modified animal models to study NDCs, and research using these models plays a vital role in developing our understanding of the biology underlying NDCs (basic research) and in the testing of potential drug treatments (preclinical research). However, despite extensive research, successful translation of laboratory findings to the clinic is rare (Pankevich et al., 2013; Silverman et al., 2022).

Systematic review provides a well-developed method for identifying areas of poor methodological quality or high risk of bias within research literature. Systematic review is a research method used to summarise and appraise all available evidence related to a pre-specified topic (Egger et al., 2001) and can identify areas for improvements, which might increase internal validity and research rigour and reproducibility.

Clinicians have used systematic reviews to inform evidence-based healthcare since the 1980s. Findings from such reviews have led to considerable improvements in the way clinical trials are conducted and reported (Plint et al., 2006). Over the last two decades, researchers have adopted systematic review methods to summarise and appraise evidence from laboratory animal studies (de Vries et al., 2014) and have achieved similar success in research improvement (McCann et al., 2016; Ramirez et al., 2017). However, the fundamental differences between clinical and animal studies mean that systematic review methodologies must be appropriately adapted (Hunniford et al., 2021). The development of several tools has been instrumental in supporting researchers conducting systematic reviews and meta-analyses of animal studies, including checklists for assessing the risk of bias (Hooijmans et al., 2014) and reporting quality (Macleod et al., 2004) of laboratory animal studies, and a protocol template for systematic reviews of animal intervention studies (de Vries et al., 2015). Despite this, reporting quality of animal systematic reviews is low (Hunniford et al., 2021; Mueller et al., 2014). Currently in development is an extension to the Preferred Reporting Items for Systematic reviews and Meta Analyses (PRISMA) guidelines specifically designed for preclinical systematic reviews (PRISMA-Pre). PRISMA-Pre is a set of reporting guidelines that aim to improve the reporting quality of preclinical systematic reviews (Hunniford et al., 2021).

Here, we aim to evaluate the quantity, characteristics, and reporting quality of systematic reviews which synthesise research using genetically modified animals to model NDCs. We assessed the reporting quality of included systematic reviews using the PRISMA-Pre checklist. We were interested in (1) models with alterations in any of the 102 high-confidence genes identified via large-scale exome sequencing by Satterstrom et al. (2020) and (2) models of Fragile X syndrome (FXS), Rett syndrome (RTT), and Angelman syndrome which have alterations in the *Fmr1*, *Mecp2*, and *Ube3a* genes, respectively. These additional 3 genes are not included in the list of 102 genes; however, they are extensively researched monogenic NDCs.

This review does not synthesise evidence from existing reviews. The rationale behind this review is that by identifying the quantity of and assessing the reporting quality of existing systematic reviews in this area, we can inform guidance on how future systematic reviews within this research field should be conducted. Findings from this review have been used to inform the development of a living evidence summary of research using genetically modified animals to model NDCs, a preliminary protocol for which is available on the Open Science Framework (OSF; Digital Object Identifier [DOI]:10.17605/OSF.IO/GFTZP).

## Methods

### Protocol registration

Stage 1 of this Registered Report received peer review via Peer Community In Registered Reports (PCI-RR) and is preregistered on OSF (DOI: https://doi.org/10.17605/OSF.IO/952QK). At the time of preregistration, we had completed preliminary searches and optimised our search strategy to inform the development of our study protocol.

### Deviations from the preregistration

We have made the following changes from stage 1 of our Registered Report:

- Introduction
  - ‘characterised by’ has been changed to ‘presenting with’.
  - ‘autism spectrum condition’ has been changed to autism to align with best practices when referring to autism outlined in ‘Avoiding Ableist Language: Suggestions for Autism Researchers’ (Bottema-Beutel et al., 2021).
  - We had originally described this work as an umbrella review. However, as we do not synthesise results from the systematic reviews we have included, we thought it more appropriate to label our work as simply a review of systematic reviews. To account for this change and to improve grammar, ‘we aim to conduct an umbrella review to identify’ has been changed to ‘we aim to evaluate’.
  - Numbers (1) and (2) have been added to improve the readability of the sentence describing our genes of interest.
  - Following acceptance of our stage 1 Registered Report, we made the decision to include reviews investigating animal models with *Ube3a* gene alterations. Our rationale for this decision is that, upon searching the literature, we realised that (similar to *Fmr1* and *Mecp2*) *Ube3a* models are extensively used in NDC research and appear to be more extensively reviewed. Alterations in *Ube3a* are highly associated with the human condition Angelman syndrome. To account for this change, we added mention of Angelman syndrome to the introduction.
  - ‘identifying the quality and reporting quality’ has been changed to ‘identifying the quality of and assessing the reporting quality’ to improve clarity of our methods.
  - ‘preliminary protocol for which has been preregistered’ in relation to future work informed by this project has been changed to ‘preliminary protocol for which is available’ as our living evidence summary protocol is not an official preregistration but rather a living document in an Open Science Framework project.

- Methods
  - Systematic search dates have been added.
  - ‘databases’ has been changed to ‘data sources’ as this is a more accurate description of these resources.
  - ‘autism spectrum condition’ has been changed to ‘autism’ (see introduction deviations).
  - We have added that the full search terms and PRISMA-Pre checklist are also available in the stage 1 Registered Report.
  - Author initials are added to the methods where appropriate to attribute author contributions.
  - We planned to use in house code to retrieve full texts but in practice for this dataset found EndNote’s full text retrieval function more convenient.
  - The number of interlibrary loans we required to access all full texts has been added.
  - We added details on how SyRF displays records in a random order and reviewers are unaware of other reviewers’ decisions. As we are concerned with reporting quality in our work, we thought it appropriate to mention our own use of randomisation and blinding.
  - Angelman syndrome models have been added to the inclusion criteria (see introduction deviations).
  - Our stage 1 Registered Report did not report the complete methods we would use to search for and present PROSPERO data, only mentioning that we would contact authors of relevant PROSPERO registrations. We added these details in stage 2. Additionally, due to time constraints, we were unable to contact authors individually. However, we did search for published versions of PROSPERO registrations marked ongoing in case they had been published.

### Bibliographic search

On 23 January 2023, EW conducted a systematic literature search on three electronic data sources: PubMed including Medline (accessed via National Center for Biotechnology Information (NCBI)), Embase (accessed via Ovid), and Web of Science Core Collection.

Our search strategy includes three components: (1) broad terms related to NDCs, ID, epilepsy, and autism, and associated genes; (2) terms related to animal models, and (3) terms related to systematic reviews or meta-analyses. Terms used to identify animal models were taken from van der Mierden et al. (2022) and terms used to identify systematic reviews and meta-analyses were taken from Langendam et al. (2021). Full search terms are given in Appendix 1 and the stage 1 Registered Report.

Where citations appeared in multiple databases, EW removed duplicate versions of the citation using the Automated Systematic Search Deduplicator (ASySD) tool (Hair et al., 2023).

### Screening

We uploaded our search results, with duplicate citations removed, to the Systematic Review Facility (SyRF) platform (RRID:SCR_018907; Bahor et al., 2021) for screening, data extraction and management of records. SyRF displays records to reviewers in a random order, and reviewers were unaware of the decisions or annotations made by other reviewers, or in the case of reconciliation, unaware of which reviewer made which decision or annotation.

Two independent reviewers (EW and GC) screened each publication for inclusion, and any disagreements were reconciled by a third independent reviewer (MM). We planned one round of screening, where we screened the full texts of all studies retrieved from our searches against our inclusion and exclusion criteria, to avoid potentially excluding systematic reviews where the decision for inclusion rests on information contained in the full text but not in the abstract (Wilson et al., 2023a).

EW retrieved full text PDF files using the find full text feature in EndNote 20 (with institutional subscription) or via hand-searching. Where we could not access the full text using our institutional subscriptions, we requested the full text via interlibrary loan. In total, we used 24 interlibrary loans. Where the full texts of relevant articles were not written in English, we planned to use Google Translate. However, our search did not return any relevant non-English-language publications.

### Inclusion and exclusion criteria

Studies were screened according to the criteria outlined below:

- Study design – We only included systematic reviews or meta-analyses that include animal studies, either as a review limited to animal studies or those which included them alongside other study types (e.g. clinical studies). We excluded all other study designs.
- Animal models – We only included systematic reviews synthesising research using genetically modified animals to model NDCs where the modified gene appeared on the list of 102 genes identified via large-scale exome sequencing by Satterstrom et al. (2020); or genetically modified animal models of FXS, RTT, or Angelman syndrome; or other genetic models of NDCs characterised by ID, epilepsy, and autism. Animal models may be of any species. A diverse range of other models of NDCs are available but are not the focus of this review and will not be included.

#### Publication type

We included systematic reviews published in (1) peer-reviewed journals, (2) as conference abstracts, or (3) as preprints (where they are identified in searches). We did not search dedicated preprint servers. We placed no restriction on publication date or language.

### Data extraction

Two independent reviewers (EW and MM) conducted conduct data extraction. Discrepancies between reviewer decisions were reconciled by a third independent reviewer (ESS). We carried out data extraction using the SyRF platform and collected the following information:

#### Bibliographic data

We extracted the names of first authors, year of publication, title, and DOI of each included review.

#### Characteristics

To understand the purpose and scope of included systematic reviews, we extracted the following characteristics from each:

- The aim of each systematic review and the primary research question each review seeks to ask.
- Whether the review only included animal studies, or also included clinical or in vitro studies.
- Which animal models the review included.
- The total number of studies included in the systematic review.
- The total number of studies investigating relevant animal models.

#### Reporting quality

There are two broad approaches to evaluating the quality of systematic reviews: addressing the completeness of reporting or assessing the risks of bias arising from the approaches which were taken. Precise evaluation of the second requires completeness of the first, so these are overlapping but distinct.

Here, we assessed the completeness of reporting (reporting quality) of each included systematic review using the 46-point checklist developed by Hunniford et al. (2021). The checklist is adapted from the PRISMA guidelines for systematic reviews and is more specific to systematic reviews of animal studies. However, the checklist is not currently an official extension to PRISMA. Although it is not recommended to use the general PRISMA guidelines to assess in vivo systematic review reporting quality, the adapted checklist for preclinical systematic reviews has been designed for this purpose. There are no validated tools for assessing risks of bias in systematic reviews of in vivo research, and we will not conduct a formal assessment of risk of bias.

The checklist items are written in full in Appendix 2 and in the stage 1 Registered Report.

### PROSPERO search

In addition, we searched the PROSPERO database to identify the status of ongoing but unpublished preregistered systematic reviews. On the 25 September 2023, we searched PROSPERO using the following terms: neurodevelopment OR neurodevelopmental OR autism OR autistic OR ASD OR intellectual disability OR epilepsy. We limited this search to reviews of animal studies for human health protocols.

Two reviewers (EW and GC) screened each of the search results based on our inclusion criteria, and disagreements were reconciled by a third reviewer (ESS). For each relevant review, EW noted the data of registration, expected start date, expected end date, and current stage of the review. We had planned to contact authors of PROSPERO registrations to establish if their review has been published; however, we did not complete this due to time constraints. We did, however, search for published versions of preregistrations labelled ‘ongoing’ in the PROSPERO system.

### Data synthesis

We did not conduct a meta-analysis or perform statistical analyses on our data. We have presented a descriptive summary of the bibliographic, characteristics, and reporting quality data extracted from each included systematic review. We scored each included systematic review using the PRISMA-Pre checklist and have provided summary graphs detailing which items of the checklist each review met. In addition, we assessed which tools are currently being used to conduct systematic reviews in this field, including the tools used to screen studies (Checklist Item 6a), extract numerical data (Checklist Item 17a), and measure study quality or risk of bias (Checklist Item 19).

## Results

### Systematic search results

**Figure 1.**
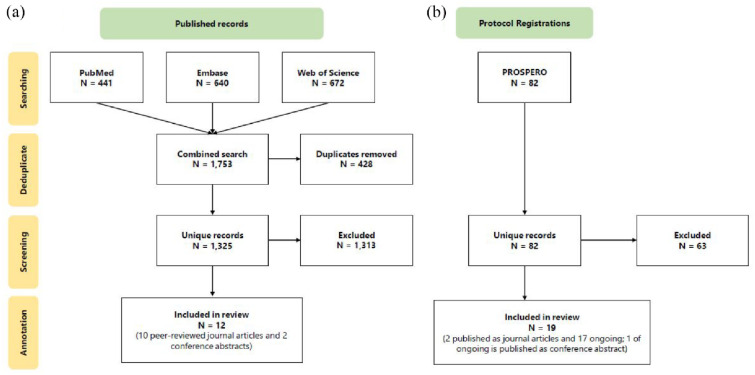
Flow chart showing the number of studies identified through searches of (a) PubMed, Embase, and Web of Science Core Collection and (b) PROSPERO, and the number of studies included after screening.

Our systematic searches of PubMed, Embase, and Web of Science Core Collection returned a total of 1,753 records (441 from PubMed, 640 from Embase, and 672 from Web of Science Core Collection; Figure 1). 428 records were removed using the ASySD tool, leaving 1,325 unique records remaining. Of these, only twelve records met our inclusion criteria via full text screening. Ten of the included records were peer-reviewed journal articles and two were conference abstracts. We did not identify any preprints through our searches. The same systematic review was identified as a conference abstract (Zhang and Spruyt, 2022) and a peer-reviewed journal article (Zhang et al., 2021). As our evaluation is primarily concerned with reporting quality of these publications, we assessed the conference abstract and journal article as separate publications.

### Characteristics of included reviews

All the reviews included in this evaluation were published between 2016 and 2022 (Table 1). Most of the reviews, including both conference abstracts, were published in 2022.

**Table 1.** Characteristics of the 12 systematic reviews included in this evaluation.

Author	Year	Journal	Publication Type	Review Type	Review Focus	Study Types	Gene Models	N Included Studies
Hardiman and Bratt	2016	*Physiol Behav*	Journal article	SR	Stress	Animal and human studies	Fmr1	17 (9)
Nakai et al.	2018	*Front Neurosci*	Journal article	SR	Neurobiology	Animal studies only	Fmr1; Mecp2; Ube3a	22 (22)
Kundap et al.	2020	*Pharmaceuticals*	Journal article	SR	Model suitability	Animal studies only	Ube3a	23 (23)
Sysoeva et al.	2020	*Clin Neurophysiol*	Journal article	SR	Biomarkers	Animal and human studies	Mecp2	28 (6)
Lyons-Warren et al.	2021	*Neurosci Biobehav Rev*	Journal article	SR	Sensory differences (olfactory)	Animal studies only	Adnp; Nrxn1; Pten; Tbr1	119 (119)
Zhang et al.^a^	2021	*J Neurosci Res*	Journal article	SR	Sleep disturbances	Animal studies only	Mecp2	13 (13)
Alamoudi et al.	2022	*Front Cell Infect Microbiol*	Journal article	SR	Microbiome	Animal and human studies	Shank3	22 (9)
Kat et al.	2022	*Neurosci Biobehav Rev*	Journal article	SR + MA	Model suitability	Animal studies only	Fmr1	266 (266)
Panzenhagen et al.	2022	*Laboratory Animals*	Conference abstract	SR + MA	Model suitability	Animal studies only	Ube3a; Pten; Shank3; Mecp2; Fmr1	531 (531)
Thawley et al.	2022	*Front Immunol*	Journal article	SR	Biomarkers	Animal studies only	Fmr1; Shank3; Pten	28 (28)
Wilde et al.	2022	*J Neurodev Disord*	Journal article	SR	Sensory differences (auditory)	Animal studies only	Adnp; Cacna2d3; Chd8; Fmr1; Mecp2; Pten; Shank3; Kcnma1; Ube3a	88 (88)
Zhang and Spruyt^a^	2022	*Sleep Medicine*	Conference abstract	SR	Sleep disturbances	Animal studies only	Mecp2	13 (13)

SR = Systematic review. MA = Meta-analysis. *N* included studies shows the total number of studies included in each review and the total number of animal studies (in brackets).

a indicates a journal article and conference abstract of the same review.

Only two reviews (a journal article and a conference abstract) conducted a meta-analysis alongside their systematic review. The remaining 10 included only qualitative synthesis of evidence.

Three of the reviews (all journal articles) included studies with animal or human participants, while the remaining reviews included only animal studies. None included in vitro or in silico work alongside animal studies.

Each of the reviews covered a distinct research question. The review aims and research questions were coded into the following topics: sleep disturbances; microbiome differences; stress (specifically the hypothalamic-pituitary-adrenal axis); neurobiology; sensory differences (specifically auditory and olfaction); model suitability; and biomarker identification (Table 1).

A variety of genetic alterations were included in the reviews evaluated, but this only represented 11 of the total 105 genetic modifications of our interest. Many reviews looked at multiple genes. The genes assessed were *Fmr1* (6 reviews); *Mecp2* (5 reviews); *Ube3a*, *Pten*, and *Shank3* (4 reviews each); *Adnp* (2 reviews); and *Cacna2d3*, *Chd8*, Kcnma1, *Nrxn1*, and *Tbr1* (1 review each). *Fmr1*, *Mecp2*, and *Ub3ea* alterations are strongly associated with FXS, RTT, and Angelman syndrome, respectively. In addition, *Pten*, *Shank3*, *Adnp*, and *Nrxn1* are associated with Cowden syndrome, Phelan-McDermid syndrome, Helsmoortel-Van der Aa syndrome, and Pitt-Hopkins-like syndrome 2, respectively (SFARI Gene, 2024). *Cacna2d3*, *Chd8*, and *Tbr1* are associated with autism and ID generally but not with any named syndromes.

The number of studies included in each review varied greatly, ranging from 13 studies to 531 (mean = 98, median = 26).

### Evaluation of reporting quality

The PRISMA-Pre checklist items are in six categories divided by the section of a manuscript which they refer to: title, introduction, methods, results, discussion, and other. A PRISMA-Pre checklist for abstracts does not yet exist, so we were mindful when evaluating the two conference abstracts and have marked reporting as not applicable for items where reporting would be unfeasible, for instance, the inclusion of a PRISMA flow diagram or summary table of included studies.

#### Items related to reporting in the title

**Figure 2.**
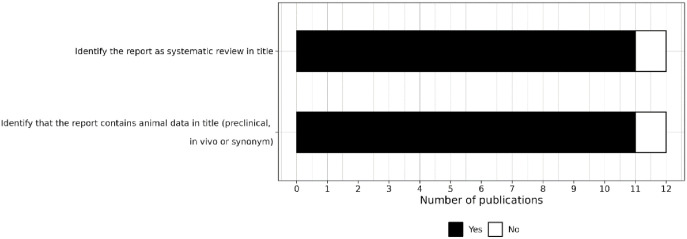
Level of reporting of items within the title section.

Eleven out of the 12 reviews identified the report as a systematic review, and 11 identified that the report contained animal data (Figure 2).

#### Items related to reporting in the introduction

**Figure 3.**
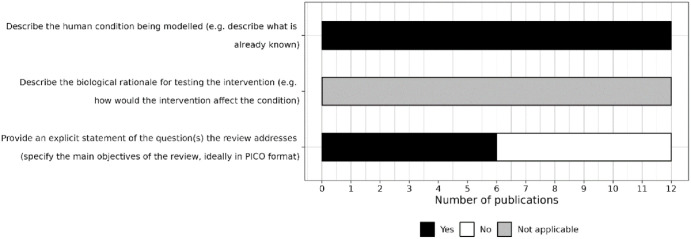
Level of reporting of items within the introduction section. PICO = Population, Intervention, Comparison, Outcome.

In the introduction section, all the reviews described the human condition being modelled, but only six reviews provided an explicit statement of the questions being addressed (Figure 3). None of the included reviews focused on the effects of interventions, so the reporting item related to describing the biological rationale for testing the intervention was not applicable for any of the reviews.

**Figure 4.**
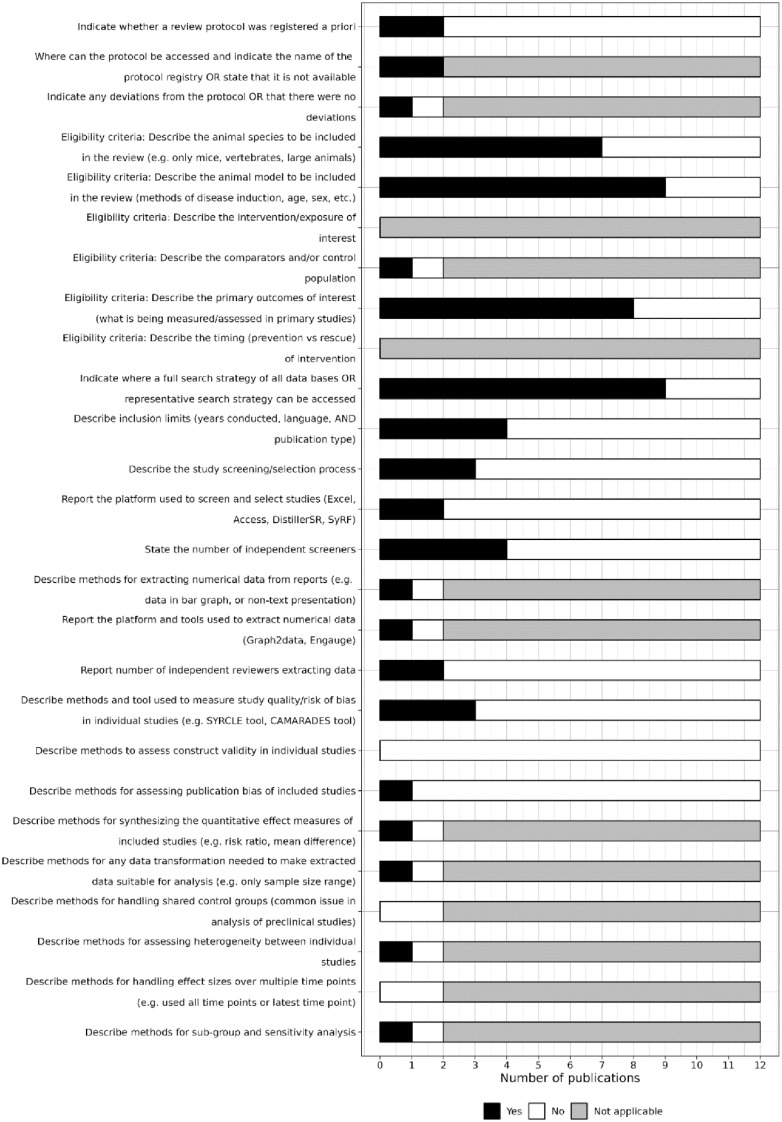
Level of reporting of items within the methods section. CAMARADES = Collaborative Approach to Meta-Analysis and Review of Animal Data from Experimental Studies. DistillerSR = Distiller Systematic Review Software. SYRCLE = SYstematic Review Centre for Laboratory animal Experimentation. SyRF = Systematic Review Facility.

Only two reviews indicated whether a protocol was registered (both were registered a priori on the PROSPERO platform). However, only one indicated whether any deviations were made to the protocol (Figure 4).

#### Items related to reporting in the methods

The eligibility criteria related to animal species, models, and outcomes of interest were reported in seven, nine, and eight of the reviews, respectively (Figure 4). Items relating to reporting interventions of interest and the timing of intervention delivery were not applicable to any of the reviews.

Nine of the 12 reviews gave the full search strategy of all databases searched, and only four described inclusion limits added to the search (Figure 4). Three reviews described the study selection process, two reported the platform used for screening (Rayyan and Microsoft Excel), four reported the number of independent screeners, and two reported the number of reviewers extracting data.

Three reviews reported methods for assessing the risk of bias of the studies they included (one used both Collaborative Approach to Meta-Analysis and Review of Animal Data from Experimental Studies (CAMARADES) and SYstematic Review Centre for Laboratory animal Experimentation (SYRCLE) tools, one used only the SYRCLE tool, and one used an unknown tool), none reported methods for construct validity assessment, and only one reported the methods for assessing publication bias (Figure 4).

Several of the reporting criteria for the methods section are related to meta-analyses, so were only applicable for the two reviews that reported a meta-analysis. One review reported eligibility criterion related to any controls or comparators, described the methods of data extraction, reported the platforms or tools used to extract numerical data (Rayyan), and described methods for synthesising the quantitative effect measures of included studies, any required data transformations, heterogeneity assessment, and sub-group or sensitivity analysis (Figure 4). The other review, which showed limited reporting, was a conference abstract and may have been affected by abstract word limits. Neither of these meta-analysis reported methods for handling shared control groups or effect sizes over multiple time points.

#### Items related to reporting in the results

**Figure 5.**
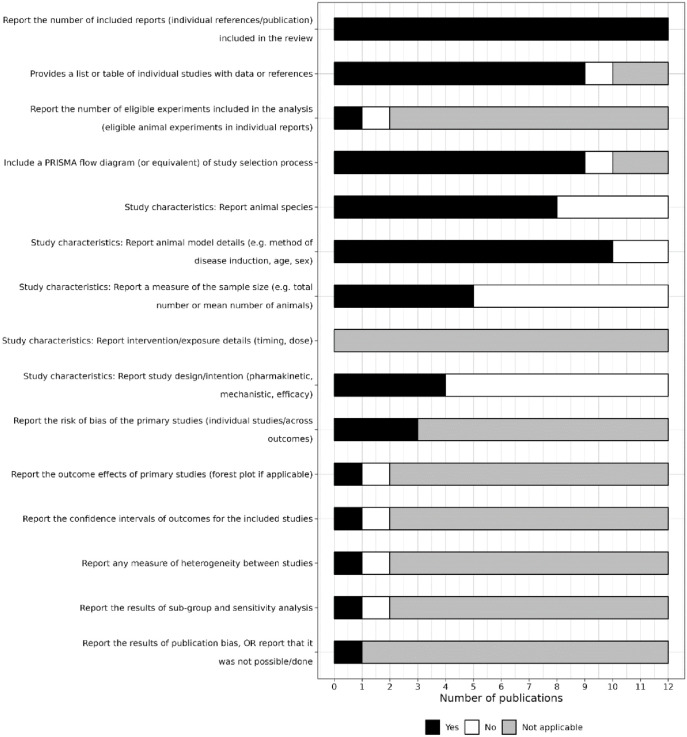
Level of reporting of items within the results section. PRISMA = Preferred Reporting Items for Systematic reviews and Meta-Analyses.

All 12 reviews reported the number of individual reports included in the review (Figure 5). Nine provided a summary table of individual studies with data and references, and nine also included a PRISMA flow diagram. These items were not applicable for the two conference abstracts. For reporting of study characteristics, 8 reviews reported the animal species, 10 reported model details, 5 reported a measure of the sample size, and 4 reported individual study designs or intentions. Reporting of intervention details was not applicable for any review. Of the three reviews which reported methods to conduct a risk of bias assessment, and one review which reported methods to analyse publication bias, all reported the results of these assessments (Figure 5).

The meta-analysis published in a peer-reviewed journal article reported the number of studies included in quantitative analysis, the outcome effects of included studies and associated confidence intervals, a measure of heterogeneity between included studies, and results from sub-group or sensitivity analysis (Figure 5). These items were not reported in the conference abstract.

#### Items related to reporting in the discussion

**Figure 6.**
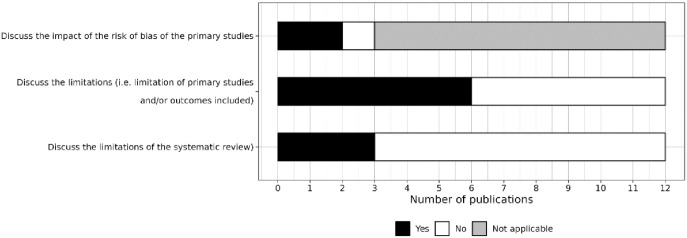
Level of reporting of items within the discussion section.

Two of the three reviews that conducted a risk of bias assessment discussed the impact of this on the included studies (Figure 6). Six reviews discussed the limitations of the individual studies they included, but only three discussed the limitations of the review itself.

#### Items related to reporting in other sections

**Figure 7.**
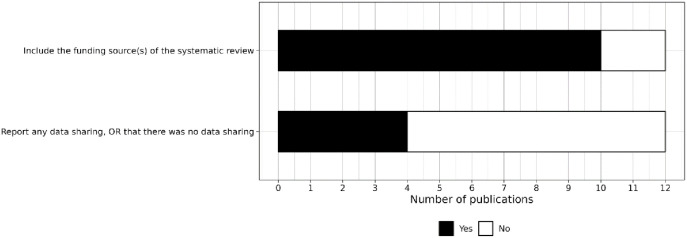
Level of reporting of items within other sections.

Finally, 10 reviews (all the journal articles) reported the source of funding for the review, and 4 reviews included a data availability statement (Figure 7).

### Identification of ongoing reviews via PROSPERO

Our search of PROSPERO identified 82 ongoing or completed reviews. After screening, we identified 19 review registrations fitting our inclusion criteria (Table 2). It was often difficult to tell if reviews would include genetic models, so we opted to be overinclusive, only excluding from our analysis if it was clear that genetic models would be excluded. Many of the protocols did not specify specific models of interest, instead often stating that they will include ‘all’ animal models of NDCs or ‘all’ animal models of autism.

**Table 2.** Anticipated start and end times, and time between each, of the 19 review registrations identified via PROSPERO.

PROSPERO ID	Anticipated Start Date	Anticipated End Date	Journal Submission Date (If Published)	Days Elapsed	Days Anticipated
CRD42018103628	16 July 2018	16 October 2018		1907	92
CRD42019122991	01 November 2018	31 August 2019		1799	303
CRD42019122981	01 November 2018	31 August 2019		1799	303
CRD42019122990	01 November 2018	31 August 2019		1799	303
CRD42019118855	01 November 2018	31 August 2019		1799	303
CRD42019122983	01 November 2018	31 August 2019		1799	303
CRD42019122988	01 November 2018	31 August 2019		1799	303
CRD42020184971	05 May 2020	30 September 2020		1248	148
CRD42020181581	08 April 2020	01 July 2020		1275	84
CRD42020191070^a^	08 June 2020	29 October 2021	18 November 2021	528	508
CRD42020196337	15 May 2020	30 April 2021		1238	350
CRD42021226299^b^	15 November 2020	31 October 2022		1054	715
CRD42022306558^a^	01 March 2021	31 March 2022	11 February 2022	347	395
CRD42022335883	01 February 2021	01 December 2022		976	668
CRD42023392578^c^	02 January 2023	01 December 2023		276	333
CRD42023382270	21 December 2022	21 April 2023		288	121
CRD42023434189	10 June 2023	01 August 2023		117	52
CRD42023425261^c^	08 May 2023	12 November 2023		150	188
CRD42023448404	10 July 2023	10 September 2023		87	62

a The registered review appears in the main review as a journal article.

b The registered review appears in the main review as a conference abstract.

c The anticipated end date has not past at the time of analysis. Time of analysis (which is also used to calculate days elapsed) was 5 October 2023.

Only two of these reviews were published at the time of our analysis; these were the two reviews from our main search which reported a protocol. The remaining 17 reviews were marked as ongoing. We did, however, identify that the unpublished conference abstract we found in our main review was registered in PROSPERO, although this registration was not reported in the conference abstract.

All the registrations were made between 2018 and 2023. Six registrations, all of which are ongoing, were made on the same date by the same first author. The mean number of days authors anticipated to complete reviews was 291 (minimum 52 days and maximum 715 days). Some of the ongoing reviews anticipated a very quick completion time (less than 100 days).

The two published reviews were registered in 2020 and 2021, and authors anticipated 508 and 395 days, respectively, to complete their reviews. In practice, they took 528 days and 347 days, respectively, from start date to journal submission (Table 2).

## Discussion

Our searches identified 10 published systematic reviews, 2 conference abstracts, and 17 ongoing reviews related to genetic animal models of NDCs. Most of the reviews are recent, with the earliest published in 2016. The first PROSPERO registration was in 2018; before 2018, PROSPERO did not allow registration of animal systematic reviews (Pieper and Rombey, 2022). We made the decision to include both Zhang et al. publications (one peer-reviewed article and one conference abstract) in our analysis, as despite the publications referring to the same systematic review research, they are separate publications and their reporting quality can be assessed separately.

From the published literature, we can see that reporting against the PRISMA-Pre checklist was mixed. Reporting in the title to identify publications as (1) systematic reviews and (2) related to animal research was overall good. One of the reasons we chose to screen based on the full text rather than one round of title and abstract screening was that we were unsure if this would cause us to erroneously exclude publications with unclear titles or abstracts.

Within the introduction section, authors consistently reported descriptions of the conditions being modelled, but only half explicitly reported the review aims. The lack of clear reporting in this area made it difficult to code the review aims in our summary table and may impact the use of individual reviews if their purpose is not clear.

Methods and results reporting were mixed. Few reviews reported having a preregistered protocol. Those that did preregister did so through PROSPERO, a dedicated repository for protocols of systematic reviews related to human health. Protocol registration is often considered a fundamental step in the systematic review process, to ensure that the review methodology is transparent and reduces the risk of bias being introduced in the systematic review (Soliman et al., 2020).

Eligibility criteria and search strategies were reported well overall, with room for improvement, while reporting of methods to screen studies was limited. The reporting of the number of studies included, and inclusion of summary tables and PRISMA flow diagrams were better. The small number of reviews that included a meta-analysis limits our ability to interpret reporting of items related to quantitative analysis with any confidence.

Few of the reviews conducted a risk of bias assessment, meaning the potential impact of risk of bias within primary studies was often not discussed. Many tools have been developed to assess the quality of evidence included in systematic reviews of animal data and associated risks of bias, including the CAMARADES checklist (Macleod et al., 2004) and the SYRCLE risk of bias tool (Hooijmans et al., 2014). Only one of the reviews that did not conduct a risk of bias assessment stated their reasoning, that the reporting of the studies they included was too poor to conduct a risk of bias assessment. However, the purpose of a risk of bias assessment is to determine precisely the reporting quality and present this evidence (Soliman et al., 2020). Systematic reviews of animal studies often observe very limited reporting quality and present that many primary studies they include are at high risk of bias (Soliman et al., 2020).

Only half of the reviews discussed the limitations of the studies they had included, and a quarter discussed the limitations of the review itself. Systematic reviews, just like any other form of research, are susceptible to research bias. Systematic reviews often play a key role in research and healthcare decision-making, so it is important that the findings of reviews are as free from bias as possible. As discussed above, protocol registration is one method for mitigating at least some of this bias and improving the transparency of the work; however, this does not negate the need to discuss limitations.

Finally, funding sources of the reviews were reported in all peer-reviewed journal articles, likely because of publishing policies. Data availability statements, which should report whether data are available or not available, were included in less than half of the reviews, despite this also being a requirement for most journals.

Systematic review is only one method used to bring understanding to translational challenges within biomedical science. In NDC research, there are many potential explanations for this translational barrier, including limitations in how research is designed, conducted, and reported (Wilson et al., 2023b), and research that is rooted in the medical model and fails to take into consideration advances in our understanding of NDCs achieved through neuroaffirmative research approaches (Heraty et al., 2023).

Systematic review of animal studies of NDCs is still an emerging activity. There are fundamental differences between clinical and animal research, such as the experimental methods used, number of participants/animals per paper, and number of relevant papers retrieved in systematic literature searches, meaning systematic review methods have to be adapted from the clinical space to fit animal research. Systematic reviews of animal research related to other biomedical conditions are ahead in this space, and many resources have been developed to help researchers conduct their reviews with rigour (de Vries et al., 2014, 2015; Hooijmans et al., 2014; Macleod et al., 2004; Sena et al., 2014). Unfortunately, our findings demonstrate that study authors of the reviews we have included have not engaged with much of this guidance and the available resources.

Systematic reviews are an empirical form of research, and if we are to rely on their findings, they must be conducted rigorously (Sena et al., 2014). When assessing the quality of research, including research like systematic reviews, we cannot directly assess the methodological quality; therefore, we assess the reporting quality as a proxy. Where the reporting of systematic review is limited, our understanding of the review’s methodological quality is limited, and in turn we have less certainty around the reliability of the review’s findings. We identified 17 systematic reviews in progress, demonstrating that this is an area of research growth. Moving forward, we encourage authors to make use of existing resources to maximise the value and impact of their work. Table 3 summarises some key resources for conducting systematic reviews of animal studies.

**Table 3.** Resources for conducting systematic reviews of animal studies.

Resource	Type	Reference or URL
Systematic reviews and meta-analysis of preclinical studies: why perform them and how to appraise them critically	General guidance article	Sena et al., 2014
A protocol format for the preparation, registration and publication of systematic reviews of animal intervention studies	Systematic review protocol template	de Vries et al., 2015
PROSPERO	Systematic review preregistration platform	https://www.crd.york.ac.uk/prospero/
Epidemiology and reporting characteristics of preclinical systematic reviews	Systematic review reporting guidelines (PRISMA-Pre checklist)	Hunniford et al., 2021
SYRCLE’s risk of bias tool for animal studies	Assessing risk of bias in animal research (SYRCLE checklist)	Hooijmans et al., 2014
Pooling of animal experimental data reveals influence of study design and publication bias	Assessing reporting quality in animal studies (CAMARADES checklist)	Macleod et al., 2004
Systematic Review Facility (SyRF)	Systematic review screening and annotation	https://syrf.org.uk/

Most of the systematic reviews we identified were published in 2022, and likely conducted during 2020–2021. We cannot know with any certainty if laboratory closures during the COVID-19 pandemic influenced the uptick in reviews conducted and whether this activity will continue to rise. However, it is important that the reporting quality, as well as the design and conduct, of systematic reviews in this area follow the advances in systematic review methodology we see in other areas, including focal cerebral ischaemia, so that they can have similar effects on primary research direction, conduct, and policy (McCann and et al, 2016).

### Strengths and limitations of this study

A major strength of our study is that, by preregistering our study using the Registered Report format, our rationale and proposed methods have been peer-reviewed, meaning we have been able to make improvements to our proposed study design prior to beginning our research.

To maximise the sensitivity (recall) of our search, we used broad search terms related to NDCs, ID, epilepsy, and autism, in parallel with terms related to individual associated genes, and screened studies based on full text to avoid erroneously excluding systematic reviews which do not report their inclusion criteria in their abstract.

A limitation, however, is that by only searching primarily English-language databases we may have missed non-English reviews (possibly evidenced by the lack of such reviews in our results).

Although we identified protocols for ongoing reviews via PROSPERO, we did not analyse the reporting of these protocols. In addition, from our results, we know that the majority of published reviews were not preregistered, suggesting that we may be unaware of many more ongoing and unpublished reviews. We used a draft extension of the PRISMA guidelines specifically designed for systematic reviews of animal studies (PRISMA-Pre; Hunniford et al., 2021; also included in Appendix 2) to assess the reporting quality of included systematic reviews. This extension remains in draft, is not yet an official extension to PRISMA and may be subject to change. The checklist was designed to improve the reporting quality of journal articles. In hindsight, it may not have been appropriate for us to apply the checklist to conference abstracts. Conference abstracts have heavily limited word counts and their purpose is to act as short communication pieces where further details are given in presentations (oral or poster).

In addition, many aspects of the PRISMA-Pre checklist were not applicable for this data set. All the systematic reviews identified were interested in mechanistic studies, so intervention-related questions within the checklist were not relevant. In addition, only two of the reviews included a meta-analysis. An improvement to the PRISMA-Pre checklist may involve adding additional subheadings to separate the meta-analysis-related questions from ‘core’ systematic review reporting items.

## Conclusion

To conclude, recent years have seen the publication of a diverse range of systematic reviews investigating genetic animal models of NDCs. Systematic evaluation of published research can help summarise research findings and strengthen evidence most effectively when effort is taken to minimise bias within the review itself. Within our sample, the reporting of published systematic reviews is mixed. We rely on clear and transparent reporting as a proxy to determine sound methodological quality. Therefore, researchers conducting systematic reviews in this space should engage with systematic review guidance, where available, to strengthen and ensure the robustness and reliability of their reviews. Increased awareness of reporting guidelines may help authors plan and report their systematic reviews with more details to increase their transparency and reproducibility.

## Appendix 1

### Final full search terms

**Table:** Animal models search terms are taken from van der Mierden et al. (2022) and systematic review search terms are taken from Langendam et al. (2021).

NCBI PubMed search strategy	
#	Search terms
**1**	‘neurodevelopmental disorders’[MeSH] OR neurodevelopment[TiAb] OR ‘neurodevelopmental delay’[TiAb] OR intellectual disability[MeSH] OR ‘intellectual disability’[TiAb] OR ‘intellectual disabilities’[TiAb] OR epilepsy[MeSH] OR epilepsy[TiAb] OR ‘autism spectrum disorder’[MeSH] OR ASD[TiAb] OR autism[TiAb] OR autistic[TiAb]
2	‘Fragile X Syndrome’[MeSH] OR ‘fragile x mental retardation protein’[MeSH] OR ‘Fragile X Syndrome’[TiAB] OR ‘fragile x mental retardation protein’[TiAB] OR ‘FMR1’[TiAB] OR ‘Rett Syndrome’[MeSH] OR ‘Methyl-CpG-Binding Protein 2’[MeSH] OR ‘Rett Syndrome’[TiAB] OR ‘Methyl-CpG-Binding Protein 2’[TiAB] OR MECP2[TiAb]
3	‘CHD8’[TiAb] OR ‘SCN2A’[TiAb] OR ‘SYNGAP1’[TiAb] OR ‘ADNP’[TiAb] OR ‘FOXP1’[TiAb] OR ‘POGZ’[TiAb] OR ‘ARID1B’[TiAb] OR ‘SUV420H1’[TiAb] OR ‘DYRK1A’[TiAb] OR ‘SLC6A1’[TiAb] OR ‘GRIN2B’[TiAb] OR ‘PTEN’[TiAb] OR ‘SHANK3’[TiAb] OR ‘MED13L’[TiAb] OR ‘GIGYF1’[TiAb] OR ‘CHD2’[TiAb] OR ‘ANKRD11’[TiAb] OR ‘ANK2’[TiAb] OR ‘ASH1L’[TiAb] OR ‘TLK2’[TiAb] OR ‘DNMT3A’[TiAb] OR ‘DEAF1’[TiAb] OR ‘CTNNB1’[TiAb] OR ‘KDM6B’[TiAb] OR ‘DSCAM’[TiAb] OR ‘SETD5’[TiAb] OR ‘KCNQ3’[TiAb] OR ‘SRPR’[TiAb] OR ‘KDM5B’[TiAb] OR ‘WAC’[TiAb] OR ‘SHANK2’[TiAb] OR ‘NRXN1’[TiAb] OR ‘TBL1XR1’[TiAb] OR ‘MYT1L’[TiAb] OR ‘BCL11A’[TiAb] OR ‘RORB’[TiAb] OR ‘RAI1’[TiAb] OR ‘DYNC1H1’[TiAb] OR ‘DPYSL2’[TiAb] OR ‘AP2S1’[TiAb] OR ‘KMT2C’[TiAb] OR ‘PAX5’[TiAb] OR ‘MKX’[TiAb] OR ‘GABRB3’[TiAb] OR ‘SIN3A’[TiAb] OR ‘MBD5’[TiAb] OR ‘MAP1A’[TiAb] OR ‘STXBP1’[TiAb] OR ‘CELF4’[TiAb] OR ‘PHF12’[TiAb] OR ‘TBR1’[TiAb] OR ‘PPP2R5D’[TiAb] OR ‘TM9SF4’[TiAb] OR ‘PHF21A’[TiAb] OR ‘PRR12’[TiAb] OR ‘SKI’[TiAb] OR ‘ASXL3’[TiAb] OR ‘SPAST’[TiAb] OR ‘SMARCC2’[TiAb] OR ‘TRIP12’[TiAb] OR ‘CREBBP’[TiAb] OR ‘TCF4’[TiAb] OR ‘CACNA1E’[TiAb] OR ‘GNAI1’[TiAb] OR ‘TCF20’[TiAb] OR ‘FOXP2’[TiAb] OR ‘NSD1’[TiAb] OR ‘TCF7L2’[TiAb] OR ‘LDB1’[TiAb] OR ‘EIF3G’[TiAb] OR ‘PHF2’[TiAb] OR ‘KIAA0232’[TiAb] OR ‘VEZF1’[TiAb] OR ‘GFAP’[TiAb] OR ‘IRF2BPL’[TiAb] OR ‘ZMYND8’[TiAb] OR ‘SATB1’[TiAb] OR ‘RFX3’[TiAb] OR ‘SCN1A’[TiAb] OR ‘PPP5C’[TiAb] OR ‘TRIM23’[TiAb] OR ‘TRAF7’[TiAb] OR ‘ELAVL3’[TiAb] OR ‘GRIA2’[TiAb] OR ‘LRRC4C’[TiAb] OR ‘CACNA2D3’[TiAb] OR ‘NUP155’[TiAb] OR ‘KMT2E [TiAb] OR ‘NR3C2’[TiAb] OR ‘NACC1’[TiAb] OR ‘PTK7’[TiAb] OR ‘PPP1R9B’[TiAb] OR ‘GABRB2’[TiAb] OR ‘HDLBP’[TiAb] OR ‘TAOK1’[TiAb] OR ‘UBR1’[TiAb] OR ‘TEK’[TiAb] OR ‘KCNMA1’[TiAb] OR ‘CORO1A’[TiAb] OR ‘HECTD4’[TiAb] OR ‘NCOA1’[TiAb] OR ‘DIP2A’[TiAb]
4	#1 OR #2 OR #3
5	(animal experimentation[MeSH] OR models, animal[MeSH] OR Animals[MeSH:noexp] OR animal population groups [MeSH] OR chordata[MeSH Terms:noexp] OR vertebrates[MeSH Terms:noexp] OR amphibians[MeSH] OR birds[MeSH] OR fishes[MeSH] OR reptiles[MeSH] OR mammals[MeSH Terms:noexp] OR primates[MeSH Terms:noexp] OR eutheria[MeSH Terms:noexp] OR artiodactyla[MeSH] OR carnivore[MeSH] OR cephalopoda[MeSH] OR cetacea[MeSH] OR chiroptera[MeSH] OR elephants[MeSH] OR hyraxes[MeSH] OR insectivora[MeSH] OR lagomorpha[MeSH] OR marsupialia[MeSH] OR monotremata[MeSH] OR perissodactyla[MeSH] OR Proboscidea Mammal[MeSH Terms:noexp] OR rodentia[MeSH] OR scandentia[MeSH] OR sirenia[MeSH] OR cingulata[MeSH] OR haplorhini[MeSH Terms:noexp] OR strepsirhini[MeSH] OR platyrrhini[MeSH] OR tarsii[MeSH] OR catarrhini[MeSH Terms:noexp] OR cercopithecidae[MeSH] OR hylobatidae[MeSH] OR hominidae[MeSH Terms:noexp] OR gorilla gorilla[MeSH] OR pan paniscus[MeSH] OR pan troglodytes[MeSH] OR pongo[MeSH] OR ((rat[tiab] OR rats[tiab] OR animal[tiab] OR animals[tiab] OR mice[tiab] OR in vivo[tiab] OR mouse[tiab] OR rabbit[tiab] OR rabbits[tiab] OR murine[tiab] OR pig[tiab] OR pigs[tiab] OR dog[tiab] OR dogs[tiab] OR bovine[tiab] OR fish[tiab] OR vertebrate[tiab] OR vertebrates[tiab] OR cat[tiab] OR cats[tiab] OR rodent[tiab] OR rodents[tiab] OR mammal[tiab] OR mammals[tiab] OR chicken[tiab] OR chickens[tiab] OR monkey[tiab] OR monkeys[tiab] OR sheep[tiab] OR canine[tiab] OR canines[tiab] OR porcine[tiab] OR cattle[tiab] OR bird[tiab] OR birds[tiab] OR hamster[tiab] OR hamsters[tiab] OR primate[tiab] OR primates[tiab] OR cow[tiab] OR cows[tiab] OR chick[tiab] OR horse[tiab] OR horses[tiab] OR avian[tiab] OR avians[tiab] OR calf[tiab] OR swine[tiab] OR swines[tiab] OR xenopus[tiab] OR turkeys[tiab] OR bear[tiab] OR bears[tiab] OR frog[tiab] OR frogs[tiab] OR zebrafish[tiab] OR goat[tiab] OR goats[tiab] OR equine[tiab] OR calves[tiab] OR poultry[tiab] OR macaque[tiab] OR macaques[tiab] OR mole[tiab] OR moles[tiab] OR ovine[tiab] OR lamb[tiab] OR lambs[tiab] OR fishes[tiab] OR diptera[tiab] OR amphibian[tiab] OR amphibians[tiab] OR snake[tiab] OR snakes[tiab] OR ruminant[tiab] OR ruminants[tiab] OR hen[tiab] OR hens[tiab] OR piglet[tiab] OR piglets[tiab] OR feline[tiab] OR felines[tiab] OR simian[tiab] OR simians[tiab] OR laevis[tiab] OR trout[tiab] OR trouts[tiab] OR teleost[tiab] OR teleosts[tiab] OR salmon[tiab] OR salmons[tiab] OR seal[tiab] OR seals[tiab] OR bull[tiab] OR bulls[tiab] ewe[tiab] OR ewes[tiab] OR hedgehog[tiab] OR hedgehogs[tiab] OR macaca[tiab] OR macacas[tiab] OR proteus[tiab] OR pigeon[tiab] OR pigeons[tiab] OR bat[tiab] OR bats[tiab] OR duck[tiab] OR ducks[tiab] OR chimpanzee[tiab] OR chimpanzees[tiab] OR baboon[tiab] OR baboons[tiab] OR deer[tiab] OR rana[tiab] OR ranas[tiab] OR carp[tiab] OR carps[tiab] OR heifer[tiab] OR swallow[tiab] OR swallows[tiab] OR lizard[tiab] OR lizards[tiab] OR canis[tiab] OR sow[tiab] OR sows[tiab] OR cynomolgus[tiab] OR quail[tiab] OR quails[tiab] OR reptile[tiab] OR reptiles[tiab] OR turtle[tiab] OR turtles[tiab] OR buffalo[tiab] OR gerbil[tiab] OR gerbils[tiab] OR boar[tiab] OR boars[tiab] OR squirrel[tiab] OR squirrels[tiab] OR oncorhynchus[tiab] OR mus[tiab] OR toad[tiab] OR toads[tiab] OR fowl[tiab] OR fowls[tiab] OR rerio[tiab] OR danio[tiab] OR ara[tiab] OR aras[tiab] OR musculus[tiab] OR tadpole[tiab] OR tadpoles[tiab] OR mulatta[tiab] OR salmo[tiab] OR ram[tiab] OR eagle[tiab] OR eagles[tiab] OR ferret[tiab] OR ferrets[tiab] OR goldfish[tiab] OR catfish[tiab] OR whale[tiab] OR whales[tiab] OR fox[tiab] OR foxes[tiab] OR ape[tiab] OR apes[tiab] OR elephant[tiab] OR elephants[tiab] OR bos[tiab] OR marmoset[tiab] OR marmosets[tiab] OR cod[tiab] OR cods[tiab] OR shark[tiab] OR sharks[tiab] OR wolf[tiab] OR eel[tiab] OR eels[tiab] OR auratus[tiab] OR rattus[tiab] OR zebra[tiab] OR zebras[tiab] OR tilapia[tiab] OR tilapias[tiab] OR gilt[tiab] OR camel[tiab] OR camels[tiab] OR squid[tiab] OR gallus[tiab] OR marsupial[tiab] OR marsupials[tiab] OR vole[tiab] OR voles[tiab] OR fascicularis[tiab] OR ovis[tiab] OR salmonid[tiab] OR salmonids[tiab] OR tiger[tiab] OR tigers[tiab] OR dolphin[tiab] OR dolphins[tiab] OR robin[tiab] OR robins[tiab] OR carpio[tiab] OR opossum[tiab] OR opossums[tiab] OR cyprinus[tiab] OR salamander[tiab] OR salamanders[tiab] OR felis[tiab] mink[tiab] OR minks[tiab] OR swan[tiab] OR swans[tiab] OR norvegicus[tiab] OR bufo[tiab] OR torpedo[tiab] OR bass[tiab] OR lamprey[tiab] OR lampreys[tiab] OR sus[tiab] OR python[tiab] OR pythons[tiab] OR tetrapod[tiab] OR tetrapods[tiab] OR shrew[tiab] shrews[tiab] OR lion[tiab] OR lions[tiab] OR hog[tiab] OR hogs[tiab] OR songbird[tiab] OR songbirds[tiab] OR oreochromis[tiab] OR starling[tiab] OR starlings[tiab] OR caprine[tiab] OR carassius[tiab] OR owl[tiab] OR owls[tiab] OR newt[tiab] OR newts[tiab] OR papio[tiab] OR scrofa[tiab] OR hare[tiab] OR hares[tiab] OR gorilla[tiab] OR gorillas[tiab] OR flounder[tiab] OR flounders[tiab] OR goose[tiab] OR herring[tiab] OR herrings[tiab] OR therian[tiab] OR buffaloes[tiab] OR canary[tiab] OR sparrow[tiab] OR sparrows[tiab] OR microtus[tiab] OR octopus[tiab] OR troglodytes[tiab] OR tuna[tiab] OR amphibia[tiab] OR chinchilla[tiab] OR chinchillas[tiab] OR ide[tiab] OR oryzias[tiab] OR cervus[tiab] OR kangaroo[tiab] OR kangaroos[tiab] OR armadillo[tiab] OR armadillos[tiab] OR callithrix[tiab] OR pan troglodytes[tiab] OR saimiri[tiab] OR cichlid[tiab] OR cichlids[tiab] OR donkey[tiab] OR donkeys[tiab] OR bream[tiab] OR char[tiab] OR
NCBI PubMed search strategy	
#	Search terms
	chars[tiab] OR finch[tiab] OR raccoon[tiab] OR raccoons[tiab] OR bothrops[tiab] OR anguilla[tiab] OR perch[tiab] OR cricetus[tiab] OR seabird[tiab] OR seabirds[tiab] OR buck[tiab] OR bucks[tiab] OR naja[tiab] OR coturnix[tiab] OR salmonids[tiab] OR geese[tiab] OR minnow[tiab] OR minnows[tiab] OR raptor[tiab] OR raptors[tiab] OR merione[tiab] OR meriones[tiab] OR rodentia[tiab] OR elaphus[tiab] OR amniote[tiab] OR amniotes[tiab] OR elasmobranch[tiab] OR emu[tiab] OR emus[tiab] OR peromyscus[tiab] OR hominid[tiab] OR hominids[tiab] OR bubalus[tiab] OR crotalus[tiab] OR gull[tiab] OR gulls[tiab] OR anas[tiab] OR anura[tiab] OR lemur[tiab] OR lemurs[tiab] OR crow[tiab] OR crows[tiab] OR camelus[tiab] OR gibbon[tiab] OR gibbons[tiab] OR waterfowl[tiab] OR parrot[tiab] OR parrots[tiab] OR eels[tiab] OR cob[tiab] OR stickleback[tiab] OR sticklebacks[tiab] OR columba[tiab] OR mesocricetus[tiab] OR ambystoma[tiab] OR raven[tiab] OR ravens[tiab] OR gadus[tiab] OR penguin[tiab] OR penguins[tiab] OR orangutan[tiab] OR orangutans[tiab] OR sturgeon[tiab] OR sturgeons[tiab] OR cuniculus[tiab] OR aves[tiab] OR virginianus[tiab] OR cephalopod[tiab] OR cephalopods[tiab] OR cebus[tiab] OR sparus[tiab] OR tortoise[tiab] OR tortoises[tiab] OR guttata[tiab] OR morhua[tiab] OR unguiculatus[tiab] OR dogfish[tiab] OR vulpes[tiab] OR mallard[tiab] OR mallards[tiab] OR apodemus[tiab] OR alligator[tiab] OR alligators[tiab] OR oryctolagus[tiab] OR llama[tiab] OR llamas[tiab] OR reindeer[tiab] OR mustela[tiab] OR duckling[tiab] OR ducklings[tiab] OR wolves[tiab] OR sander[tiab] OR amazona[tiab] OR zebu[tiab] OR badger[tiab] OR badgers[tiab] OR dove[tiab] OR doves[tiab] OR ictalurus[tiab] OR capra[tiab] OR capras[tiab] OR equus[tiab] OR camelid[tiab] OR camelids[tiab] OR poecilia[tiab] OR mule[tiab] OR mules[tiab] OR perciformes[tiab] OR salvelinus[tiab] OR labrax[tiab] OR cyprinidae[tiab] OR ariidae[tiab] OR crocodile[tiab] OR crocodiles[tiab] OR fundulus[tiab] OR dicentrarchus[tiab] OR clarias[tiab] OR cercopithecus[tiab] OR chiroptera[tiab] OR alpaca[tiab] OR alpacas[tiab] OR pike[tiab] OR pikes[tiab] OR paralichthys[tiab] OR puma[tiab] OR pumas[tiab] OR didelphis[tiab] OR pisces[tiab] OR macropus[tiab] OR triturus[tiab] OR bison[tiab] OR bisons[tiab] OR epinephelus[tiab] OR gasterosteus[tiab] OR panthera[tiab] OR acipenser[tiab] OR mackerel[tiab] OR mackerels[tiab] OR tamarin[tiab] OR tamarins[tiab] OR ostrich[tiab] OR anolis[tiab] OR vervet[tiab] OR vervets[tiab] OR wallaby[tiab] OR glareolus[tiab] OR beaver[tiab] OR beavers[tiab] OR dromedary[tiab] OR catus[tiab] OR killifish[tiab] OR pimephales[tiab] OR promelas[tiab] OR aotus[tiab] OR phoca[tiab] OR panda[tiab] OR pandas[tiab] OR porpoise[tiab] OR porpoises[tiab] OR myotis[tiab] OR yak[tiab] OR yaks[tiab] OR agkistrodon[tiab] OR vipera[tiab] OR otter[tiab] OR otters[tiab] OR turbot[tiab] OR turbots[tiab] OR squamate[tiab] OR carnivora[tiab] OR mullet[tiab] OR mullets[tiab] OR hawk[tiab] OR hawks[tiab] OR taeniopygia[tiab] OR seahorse[tiab] OR seahorses[tiab] OR poecilia reticulata[tiab] OR falcon[tiab] OR falcons[tiab] OR prosimian[tiab] OR prosimians[tiab] OR parus[tiab] OR perca[tiab] OR fingerling[tiab] OR fingerlings[tiab] OR antelope[tiab] OR antelopes[tiab] OR tupaia[tiab] OR passeriformes[tiab] OR sepia[tiab] OR saguinus[tiab] OR coyote[tiab] OR coyotes[tiab] OR pongo[tiab] OR meleagris[tiab] OR reptilia[tiab] OR lepus[tiab] OR psittacine[tiab] OR hagfish[tiab] OR warbler[tiab] OR warblers[tiab] OR russell’s viper[tiab] OR russell’s vipers[tiab] OR smolt[tiab] OR smolts[tiab] OR budgerigar[tiab] OR sardine[tiab] OR sardines[tiab] OR cavia[tiab] OR cavias[tiab] OR hyla[tiab] OR pleurodeles[tiab] OR siluriformes[tiab] OR great tit[tiab] OR great tits[tiab] OR guppy[tiab] OR bonobo[tiab] OR bonobos[tiab] OR rutilus[tiab] OR trichosurus[tiab] OR muridae[tiab] OR phodopus[tiab] OR channa[tiab] OR squalus[tiab] OR lynx[tiab] OR sturnus[tiab] OR petromyzon[tiab] OR vitulina[tiab] OR monodelphis[tiab] OR cuttlefish[tiab] OR adder[tiab] OR adders[tiab] OR lepomis[tiab] OR canaria[tiab] OR gambusia[tiab] OR guppies[tiab] OR xiphophorus[tiab] OR flatfish[tiab] OR koala[tiab] OR koalas[tiab] OR labeo[tiab] OR stingray[tiab] OR stingrays[tiab] OR chelonia[tiab] OR lampetra[tiab] OR spermophilus[tiab] OR crocodilian[tiab] OR passer domesticus[tiab] OR sciurus[tiab] OR artiodactyla[tiab] OR ranidae[tiab] OR corvus[tiab] OR necturus[tiab] OR platypus[tiab] OR canaries[tiab] OR bovid[tiab] OR lagopus[tiab] OR trimeresurus[tiab] OR gariepinus[tiab] OR marten[tiab] OR martens[tiab] OR drosophilidae[tiab] OR mugil[tiab] OR sunfish[tiab] OR porcellus[tiab] OR cypriniformes[tiab] OR alouatta[tiab] OR scophthalmus[tiab] OR anser[tiab] OR electrophorus[tiab] OR putorius[tiab] OR iguana[tiab] OR iguanas[tiab] OR lama[tiab] OR lamas[tiab] OR takifugu[tiab] OR circus[tiab] OR eptesicus[tiab] OR flycatcher[tiab] OR galago[tiab] OR galagos[tiab] OR trachemys[tiab] OR lungfish[tiab] OR characiformes[tiab] OR shorebird[tiab] OR shorebirds[tiab] OR giraffe[tiab] OR giraffes[tiab] OR micropterus[tiab] OR scyliorhinus[tiab] OR cichlidae[tiab] OR loligo[tiab] OR porcupine[tiab] OR porcupines[tiab] OR chub[tiab] OR chubs[tiab] OR solea[tiab] OR pleuronectes[tiab] OR hylidae[tiab] OR viperidae[tiab] OR echis[tiab] OR sorex[tiab] OR anchovy[tiab] OR lagomorph[tiab] OR ostriches[tiab] OR vulture[tiab] OR vultures[tiab] OR whitefish[tiab] OR araneus[tiab] OR jird[tiab] OR jirds[tiab] OR tern[tiab] OR esox[tiab] OR drake[tiab] OR drakes[tiab] OR elapidae[tiab] OR gallopavo[tiab] OR chordata[tiab] OR myodes[tiab] OR caretta[tiab] OR serinus[tiab] OR grouse[tiab] OR misgurnus[tiab] OR meles[tiab] OR blackbird[tiab] blackbirds[tiab] OR coregonus[tiab] OR bobwhite[tiab] OR bobwhites[tiab] OR heteropneustes[tiab] OR mammoth[tiab] OR mammoths[tiab] OR turdus[tiab] OR rhinella[tiab] OR ateles[tiab] OR characidae[tiab] OR clupea[tiab] OR bungarus [tiab] OR brill[tiab] OR struthio camelus[tiab] OR sloth[tiab] OR sloths[tiab] OR pteropus[tiab] OR sculpin[tiab] OR anthropoids[tiab] OR pollock[tiab] OR pollocks[tiab] OR morone[tiab] OR pan paniscus[tiab] OR litoria[tiab] OR chipmunk[tiab] OR chipmunks[tiab] OR balaenoptera[tiab] OR marmota[tiab] OR melopsittacus[tiab] OR hyrax[tiab] OR lemming[tiab] OR lemmings[tiab] OR halibut[tiab] OR hylobates[tiab] OR lates[tiab] OR caiman[tiab] OR caimans[tiab] OR sigmodon[tiab] OR stenella[tiab] OR barbel[tiab] OR barbels[tiab] OR sterna[tiab] OR parakeet[tiab] OR parakeets[tiab] OR phocoena[tiab] OR leptodactylus[tiab] OR canidae[tiab] OR buteo[tiab] OR harengus[tiab] OR gopher[tiab] OR gophers[tiab] OR marmot[tiab] OR marmots[tiab] OR gosling[tiab] OR goslings[tiab] OR platichthys[tiab] OR gar[tiab] OR gars[tiab] OR sebastes[tiab] OR marsupialia[tiab] OR notophthalmus[tiab] OR gazelle[tiab] OR gazelles[tiab] OR insectivora[tiab] OR paridae[tiab] OR felidae[tiab] OR russula[tiab] OR galliformes[tiab] OR bombina[tiab] OR colobus [tiab] OR echidna[tiab] OR echidnas[tiab] OR seabass[tiab] OR syncerus[tiab] OR plaice[tiab] OR blue tit[tiab] OR blue tits[tiab] OR pagrus[tiab] OR catfishes[tiab] OR cetacea[tiab] OR barbus[tiab] OR cygnus[tiab] OR ficedula[tiab] OR chamois[tiab] OR colubridae[tiab] OR perches[tiab] OR coelacanth[tiab] OR fitch[tiab] OR urodela[tiab] OR cynops[tiab] OR martes[tiab] OR halichoerus[tiab] OR aix[tiab] OR salmonidae[tiab] OR leuciscus[tiab] OR magpie[tiab] OR magpies[tiab] OR silurus[tiab] OR whiting[tiab] OR whitings[tiab] OR anseriformes[tiab] OR colinus[tiab] OR rhea[tiab] OR chlorocebus[tiab] OR octodon[tiab] OR acinonyx[tiab] OR mouflon[tiab] OR mouflons[tiab] OR ibex[tiab] OR tetraodon[tiab] OR bufonidae[tiab] OR equidae[tiab] OR jackal[tiab] OR cephalopoda[tiab] OR dendroaspis[tiab] OR glama[tiab] OR muskrat[tiab] OR muskrats[tiab] OR sable[tiab] OR sables[tiab] OR wildebeest[tiab] OR streptopelia[tiab] OR albifrons[tiab] OR vespertilionidae[tiab] OR woodpecker[tiab] OR woodpeckers[tiab] OR muntjac[tiab] OR muntjacs[tiab] OR archosaur[tiab] OR branta[tiab] OR cricetulus[tiab] OR megalobrama[tiab] OR poeciliidae[tiab] OR desmodus[tiab] OR snakehead[tiab] OR snakeheads[tiab] OR tench[tiab] OR teal[tiab] OR teals[tiab] OR bandicoot[tiab] OR bandicoots[tiab] OR apteronotus[tiab] OR phyllostomidae[tiab] OR crocidura[tiab] OR buzzard[tiab] OR buzzards[tiab] OR larimichthys[tiab] OR cercocebus[tiab] OR pipistrellus[tiab] OR erithacus[tiab] OR impala[tiab] OR impalas[tiab] OR rousettus[tiab] OR haddock[tiab] OR haddocks[tiab] OR tinca[tiab] OR ratite[tiab] OR calidris[tiab] OR cynoglossus[tiab] OR hypophthalmichthys[tiab] OR bullock[tiab] OR bullocks[tiab] OR dromedaries[tiab] OR alectoris[tiab] OR filly[tiab] OR salamandra[tiab] OR cingulata[tiab] OR bitis[tiab] OR grus[tiab] OR ammodytes[tiab] OR macaw[tiab] OR macaws[tiab] OR hypoleuca[tiab] OR sapajus[tiab] OR cyprinodontiformes[tiab] OR hippopotamus[tiab] OR pelophylax[tiab] OR capybara[tiab] OR capybaras[tiab] OR weasel[tiab] OR weasels[tiab] OR cairina[tiab] OR cynomys[tiab] OR lutra[tiab] OR cockatoo[tiab] OR cockatoos[tiab] OR lachesis[tiab] OR lagomorpha[tiab] OR rupicapra[tiab] OR daboia[tiab] OR orang utan[tiab] OR orang utans[tiab] OR platyrrhini[tiab] OR charadriiformes[tiab] OR micrurus[tiab] OR psittaciformes[tiab] OR spalax[tiab] OR loris[tiab] OR mustelidae[tiab] OR sylvilagus[tiab] OR vitticeps[tiab] OR cockatiel[tiab] OR mustelus[tiab] OR cottus[tiab] OR erythrocebus[tiab] OR dipodomys[tiab] OR platessa[tiab] OR callicebus[tiab] OR loricariidae[tiab] OR catostomus[tiab] OR cuneata[tiab] OR cyanistes[tiab] OR cyprinodon[tiab] OR sigmodontinae[tiab] OR
NCBI PubMed search strategy	
#	Search terms
	elasmobranchii[tiab] OR trichechus[tiab] OR sauropsid[tiab] OR xenarthra[tiab] OR dormouse[tiab] OR perissodactyla[tiab] OR nautilus[tiab] OR cirrhinus[tiab] OR gulo[tiab] OR tragelaphus[tiab] OR merula[tiab] OR numida[tiab] OR sciaenidae[tiab] OR cerastes[tiab] OR sciuridae[tiab] OR gibbosus[tiab] OR octopuses[tiab] OR eland[tiab] OR elands[tiab] OR phyllomedusa[tiab] OR pogona[tiab] OR walrus[tiab] OR agamidae[tiab] OR leptodactylidae[tiab] OR ridibundus[tiab] OR leontopithecus[tiab] OR anteater[tiab] OR anteaters[tiab] OR pelodiscus[tiab] OR cebidae[tiab] OR columbianus[tiab] OR pelteobagrus fulvidraco[tiab] OR hominoidea[tiab] OR mandrillus[tiab] OR zonotrichia leucophrys[tiab] OR agama[tiab] OR gobiocypris[tiab] OR bearded dragon[tiab] OR bearded dragons[tiab] OR sarotherodon[tiab] OR talpa[tiab] OR discoglossus[tiab] OR hagfishes[tiab] OR sphenodon[tiab] OR gudgeon[tiab] OR amphiuma[tiab] OR aythya[tiab] OR tenrec[tiab] OR tenrec[tiab] OR hominidae[tiab] OR risoria[tiab] OR salamandridae[tiab] OR camelidae[tiab] OR columbiformes[tiab] OR latimeria[tiab] OR plover[tiab] OR plovers[tiab] OR afrotheria[tiab] OR falco sparverius[tiab] OR polecat[tiab] OR polecats[tiab] OR crotalinae[tiab] OR salvadora[tiab] OR tarsier[tiab] OR lucioperca[tiab] OR anchovies[tiab] OR lungfishes[tiab] OR terrapin[tiab] OR dromaius novaehollandiae[tiab] OR lateolabrax[tiab] OR eigenmannia[tiab] OR pelamis[tiab] OR theropithecus[tiab] OR murinae[tiab] OR gander[tiab] OR gymnotus[tiab] OR pseudacris[tiab] OR gymnophiona[tiab] OR gymnotiformes[tiab] OR laticauda[tiab] OR falconiformes[tiab] OR dugong[tiab] OR dugongs[tiab] OR pintail[tiab] OR pintails[tiab] OR rook[tiab] OR rooks[tiab] ORl asiurus[tiab] OR catshark[tiab] OR catsharks[tiab] OR micropogonias[tiab] OR red junglefowl[tiab] OR paddlefish[tiab] OR ophiophagus[tiab] OR hollandicus[tiab] OR nymphicus[tiab] OR pimelodidae[tiab] OR aepyceros[tiab] OR cobitidae[tiab] OR strigiformes[tiab] OR cobitis[tiab] OR dormice[tiab] OR alytes[tiab] OR calloselasma[tiab] OR guanaco[tiab] OR phasianidae[tiab] OR round goby[tiab] OR trichogaster[tiab] OR catarrhini[tiab] OR eelpout[tiab] OR eelpouts[tiab] OR galaxias[tiab] OR gaur[tiab] OR pungitius[tiab] OR suslik[tiab] OR susliks[tiab] OR flatfishes[tiab] OR percidae[tiab] OR caprinae[tiab] OR todarodes[tiab] OR osmerus[tiab] OR ameiurus[tiab] OR anthropoidea[tiab] OR castor canadensis[tiab] OR pouting[tiab] OR poutings[tiab] OR tetraodontiformes[tiab] OR arvicolinae[tiab] OR siamang[tiab] OR siamangs[tiab] OR castor fiber[tiab] OR nomascus[tiab] OR red knot[tiab] OR red knots[tiab] OR syngnathidae[tiab] OR iguanidae[tiab] OR eretmochelys[tiab] OR ursidae[tiab] OR callimico[tiab] OR columbidae[tiab] OR microhylidae[tiab] OR anaxyrus[tiab] OR menidia[tiab] OR pipistrelle[tiab] OR greylag[tiab] OR pipidae[tiab] OR scandentia[tiab] OR bowfin[tiab] OR bowfins[tiab] OR dendrobatidae[tiab] OR zenaida[tiab] OR bushbaby[tiab] OR harrier[tiab] OR harriers[tiab] OR macropodidae[tiab] OR pygerythrus[tiab] OR clupeidae[tiab] OR odorrana[tiab] OR corvidae[tiab] OR jerboa[tiab] OR jerboas[tiab] OR canutus[tiab] OR hylobatidae[tiab] OR clupeiformes[tiab] OR great cormorant[tiab] OR great cormorants[tiab] OR scorpaeniformes[tiab] OR chondrostean[tiab] OR garfish[tiab] OR proboscidea[tiab] OR psetta[tiab] OR diapsid[tiab] OR serotinus[tiab] OR tetrao[tiab] OR walruses[tiab] OR carcharhiniformes[tiab] OR leucoraja[tiab] OR pumpkinseed[tiab] OR dosidicus[tiab] OR acipenseriformes[tiab] OR daubentonii[tiab] OR emberizidae[tiab] OR gadiformes[tiab] OR hyraxes[tiab] OR stizostedion[tiab] OR wolverine[tiab] OR wolverines[tiab] OR lissotriton[tiab] OR acanthurus[tiab] OR centrarchidae[tiab] OR gloydius[tiab] OR laurasiatheria[tiab] OR limosa[tiab] OR psittacula[tiab] OR leporidae[tiab] OR proteidae[tiab] OR zander[tiab] OR zanders[tiab] OR arapaima[tiab] OR bagridae[tiab] OR cyprinodontidae[tiab] OR mithun[tiab] OR pandion[tiab] OR jackdaw[tiab] OR jackdaws[tiab] OR procyonidae[tiab] OR carus[tiab] OR jaculus[tiab] OR salmoniformes[tiab] OR common sole[tiab] OR common soles[tiab] OR protobothrops[tiab] OR calamita[tiab] OR brachyteles[tiab] OR trionyx[tiab] OR turdidae[tiab] OR boidae[tiab] OR luscinia[tiab] OR pugnax[tiab] OR euarchontoglires[tiab] OR saithe[tiab] OR saithes[tiab] OR symphalangus[tiab] OR aardvark[tiab] OR aardvarks[tiab] OR oystercatcher[tiab] OR oystercatchers[tiab] OR arius[tiab] OR corydoras[tiab] OR poacher[tiab] OR poachers[tiab] OR aurochs[tiab] OR cebuella[tiab] OR crecca[tiab] OR lemuridae[tiab] OR sirenia[tiab] OR lemmus[tiab] OR perdix[tiab] OR glires[tiab] OR lepidosaur[tiab] OR muskox[tiab] OR deinagkistrodon[tiab] OR pholidota[tiab] OR holocephali[tiab] OR cercopithecinae[tiab] OR clariidae[tiab] OR agapornis[tiab] OR doryteuthis[tiab] OR tyrannidae[tiab] OR dicroglossidae[tiab] OR godwit[tiab] OR godwits[tiab] OR monedula[tiab] OR pongidae[tiab] OR atheriniformes[tiab] OR colobinae[tiab] OR lophocebus[tiab] OR atelidae[tiab] OR cottidae[tiab] OR leucopsis[tiab] OR acanthuridae[tiab] OR didelphimorphia[tiab] OR elver[tiab] OR elvers[tiab] OR lapponica[tiab] OR dermoptera[tiab] OR european hake[tiab] OR european hakes[tiab] OR gerbillinae[tiab] OR banteng[tiab] OR hartebeest[tiab] OR hartebeests[tiab]OR hogget[tiab] OR haematopus[tiab] OR anguis fragilis[tiab] OR grey heron[tiab] OR grey herons[tiab] OR blue whiting[tiab] OR blue whitings[tiab] OR furnariidae[tiab] OR macrovipera[tiab] OR esocidae[tiab] OR lapwing[tiab] OR lapwings[tiab] OR mylopharyngodon[tiab] OR wallabia[tiab] OR beloniformes[tiab] OR potoroo[tiab] OR potoroos[tiab] OR athene noctua[tiab] OR pleuronectidae[tiab] OR bushbabies[tiab] OR muscicapidae[tiab] OR alligatoridae[tiab] OR fuligula[tiab] OR bush baby[tiab] OR guineafowl[tiab] OR spoonbill[tiab] OR spoonbills[tiab] OR viverridae[tiab] OR catostomidae[tiab] OR zebrafishes[tiab] OR ibexes[tiab] OR vendace[tiab] OR estrildidae[tiab] OR monotremata[tiab] OR sepiella[tiab] OR ambystomatidae[tiab] OR shelduck[tiab] OR shelducks[tiab] OR treeshrew[tiab] OR treeshrews[tiab] OR hoplobatrachus[tiab] OR pochard[tiab] OR hoolock[tiab] OR hoolocks[tiab] OR lynxes[tiab] OR antilope[tiab] OR antilopes[tiab] OR blackbuck[tiab] OR blackbucks[tiab] OR cricetinae[tiab] OR paramisgurnus[tiab] OR skylark[tiab] OR skylarks[tiab] OR soleidae[tiab] OR allobates[tiab] OR northern wheatear[tiab] OR northern wheatears[tiab] OR pitheciidae[tiab] OR takin[tiab] OR theria[tiab] OR vanellus[tiab] OR galaxiidae[tiab] OR lorisidae[tiab] OR ostralegus[tiab] OR palaeognathae[tiab] OR stone loach[tiab] OR alauda[tiab] OR callitrichinae[tiab] OR caniformia[tiab] OR duttaphrynus[tiab] OR ictaluridae[tiab] OR osteoglossiformes[tiab] OR poultries[tiab] OR curema[tiab] OR ruddy turnstone[tiab] OR ruddy turnstones[tiab] OR sheatfish[tiab] OR sunfishes[tiab] OR centropomidae[tiab] OR hemachatus[tiab] OR platalea[tiab] OR thamnophilidae[tiab] OR song thrush[tiab] OR atherinopsidae[tiab] OR siluridae[tiab] OR tadorna[tiab] OR chroicocephalus[tiab] OR ermine[tiab] OR ermines[tiab] OR gavialis[tiab] OR ruff[tiab] OR tupaiidae[tiab] OR diprotodontia[tiab] OR hyaenidae[tiab] OR antilopinae[tiab] OR crocodylidae[tiab] OR herpestidae[tiab] OR hippopotamidae[tiab] OR northern shoveler[tiab] OR round gobies[tiab] OR cheirogaleidae[tiab] OR indriidae[tiab] OR fundulidae[tiab] OR pythonidae[tiab] OR rhynchocephalia[tiab] OR anodorhynchus[tiab] OR red-backed shrike[tiab] OR red-backed shrikes[tiab] OR triakidae[tiab] OR phalangeridae[tiab] OR aoudad[tiab] OR boreoeutheria[tiab] OR eurasianjay[tiab] OR eurasian jays[tiab] OR feliformia[tiab] OR haplorhini[tiab] OR osteoglossidae[tiab] OR paenungulata[tiab] OR struthioniformes[tiab] OR ferina[tiab] OR sanderling[tiab] OR sanderlings[tiab] OR spheniscidae[tiab] OR cuttlefishes[tiab] OR cygnet[tiab] OR dasycneme[tiab] OR gadwall[tiab] OR gadwalls[tiab] OR pelobates fuscus[tiab] OR wryneck[tiab] OR wrynecks[tiab] OR afrosoricida[tiab] OR culaea[tiab] OR dover sole[tiab] OR dover soles[tiab] OR paralichthyidae[tiab] OR passeridae[tiab] OR osteolaemus[tiab] OR song thrushes[tiab] OR bluethroat[tiab] OR bluethroats[tiab] OR hydrophiidae[tiab] OR megrim[tiab] OR mephitidae[tiab] OR strepsirhini[tiab] OR tomistoma[tiab] OR epidalea[tiab] OR osmeriformes[tiab] OR bush babies[tiab] OR tarsiiform[tiab] OR atelinae[tiab] OR bufotes[tiab] OR eurasian coot[tiab] OR eurasian coots[tiab] OR galagidae[tiab] OR geopelia[tiab] OR philomachus[tiab] OR tubulidentata[tiab] OR bombinatoridae[tiab] OR pelobatidae[tiab] OR tachysurus[tiab] OR ailuridae[tiab] OR woodlark[tiab] OR woodlarks[tiab] OR alcelaphinae[tiab] OR redshank[tiab] OR redshanks[tiab] OR salientia[tiab] OR sand smelt[tiab] OR sand smelts[tiab] OR woodmice[tiab] OR woodmouse[tiab] OR dasyproctidae[tiab] OR eurasian wigeon[tiab] OR eurasian wigeons[tiab]OR garganey[tiab] OR garganeys[tiab] OR lemon sole[tiab] OR lemon soles[tiab] OR common dab[tiab] OR common dabs[tiab] OR graylag[tiab] OR graylags[tiab] OR leucorodia[tiab] OR osphronemidae[tiab] OR bewickii[tiab] OR common moorhen[tiab] OR common moorhens[tiab] OR decapodiformes[tiab] OR gobbler[tiab] OR gobblers[tiab] OR odontophoridae[tiab] OR paddlefishes[tiab] OR eutheria[tiab] OR salmonine[tiab] OR esociformes[tiab] OR eurasian woodcock[tiab] OR eurasian woodcocks[tiab] OR european smelt[tiab] OR european smelts[tiab] OR goldfishes[tiab] OR tenches[tiab] OR tyranni[tiab] OR common chaffinch[tiab] OR common chaffinchs[tiab] OR common redstart[tiab] OR common redstarts[tiab] OR
NCBI PubMed search strategy	
#	Search terms
	common roach[tiab] OR common roachs[tiab] OR great knot[tiab] OR great knots[tiab] OR potoroidae[tiab] OR alytidae[tiab] OR coregonine[tiab] OR dipteral[tiab] OR leveret[tiab] OR poeciliopsis gracilis[tiab] OR amphiumidae[tiab] OR batrachoidiformes[tiab] OR bighead goby[tiab] OR heteropneustidae[tiab] OR lullula[tiab] OR norway pout[tiab] OR norway pouts[tiab] OR sipunculida[tiab] OR dogfishes[tiab] OR sebastidae[tiab] OR tarsiidae[tiab] OR alethinophidia[tiab] OR common nase[tiab] OR common nases[tiab] OR common sandpiper[tiab] OR common sandpipers[tiab] OR eurasian blackcap[tiab] OR eurasian blackcaps[tiab] OR pterocnemia[tiab] OR syngnathiformes[tiab] OR common chaffinches[tiab] OR eupleridae[tiab] OR octopodiformes[tiab] OR phascolarctidae[tiab] OR scophthalmidae[tiab] OR starry smooth-hound[tiab] OR starry smooth-hounds[tiab] OR whitefishes[tiab] OR cuniculidae[tiab] OR european sprat[tiab] OR european sprats[tiab] OR rosy bitterling[tiab] OR rosy bitterlings[tiab] OR common dace[tiab] OR common daces[tiab] OR lesser weever[tiab] OR lesser weevers[tiab] OR scaldfish[tiab] OR water rail[tiab] OR water rails[tiab] OR alouattinae[tiab] OR centrarchiformes[tiab] OR common whitethroat[tiab] OR common whitethroats[tiab] OR gavialidae[tiab] OR grey gurnard[tiab] OR grey gurnards[tiab] OR lateolabracidae[tiab] OR rheiformes[tiab] OR tubgurnard[tiab] OR tub gurnards[tiab] OR common chiffchaff[tiab] OR common chiffchaffs[tiab] OR garfishes[tiab] OR lesser whitethroat[tiab] OR lesser whitethroats[tiab] OR myoxidae[tiab] OR seabasses[tiab] OR spariformes[tiab] OR umbridae[tiab] OR yellow boxfish[tiab] OR anabantiformes[tiab] OR aotidae[tiab] OR common bleak[tiab] OR common bleaks[tiab] OR common rudd[tiab] OR common rudds[tiab] OR greater pipefish[tiab] OR hapale[tiab] OR nandiniidae[tiab] OR stone loaches[tiab] OR whinchat[tiab] OR whinchats[tiab] OR acanthuriformes[tiab] OR brotula barbata[tiab] OR common ling[tiab] OR common lings[tiab] OR common roaches[tiab] OR cottonrat[tiab] OR cottonrats[tiab] OR douroucoulis[tiab] OR dromaiidae[tiab] OR fitches[tiab] OR fitchew[tiab] OR galaxiiformes[tiab] OR laprine[tiab] OR saimiriinae[tiab] OR solenette[tiab] OR tarsii[tiab] OR tompot blenny[tiab] OR common dragonet[tiab] OR common dragonets[tiab] OR longspined bullhead[tiab] OR longspined bullheads[tiab] OR monotremate[tiab] OR monotremates[tiab] OR pempheriformes[tiab] OR perdicinae[tiab] OR presbytini[tiab] OR smegmamorpha[tiab] OR bighead gobies[tiab] OR carangaria incertae sedis[tiab] OR coiidae[tiab] OR fivebeard rockling[tiab] OR foulmart[tiab] OR foumart[tiab] OR grasskeet[tiab] OR greater pipefishes[tiab] OR ibices[tiab] OR millionfish[tiab] OR muguliformes[tiab] OR norwegian topknot[tiab] OR peewit[tiab] OR red sea sailfin tang[tiab] OR rupicapras[tiab] OR sheatfishes[tiab] OR tompot blennies[tiab] OR twait shad[tiab] OR yellow boxfishes[tiab] NOT medline(sb)
6	4 AND 5
7	((systematic review[tiab] OR systematic reviews[tiab] OR meta-analyses[tiab] OR meta-analysis[tiab] OR metaanalyses[tiab] OR metaanalysis[tiab] OR systematic literature review[tiab] OR comprehensive literature review[tiab] OR Systematic survey[tiab] OR systematic overview[tiab] OR ‘Syst Rev’(Journal) OR meta-analysis(pt) OR Systematically review[tiab] OR Systematically searched[tiab] OR Systematic search[tiab] OR systematic-literature-search*[tiab] OR Meta synthesis[tiab] OR PRISMA[tiab] OR ((electronic-database*[tiab] OR databases-search*[tiab] OR electronic-search*[tiab] OR comprehensive-search*[tiab] OR literature review[tiab] OR literature search[tiab] OR literature searches[tiab] OR literature searching[tiab] OR data collection[tiab] AND (Pubmed[tiab] OR Medline[tiab] OR Embase[tiab] OR study-selection[tiab] OR selection-criteri*[tiab] OR Web of Science[tiab] OR Google[tiab] OR Scopus[tiab] OR BIOSIS[tiab] NOT (letter(pt) OR newspaper article(pt) OR comment(pt)
8	6 AND 7
Ovid Embase search strategy	
#	Search terms
1	((neurodevelop* OR neurodevelop* delay* OR intellectual disabilit* OR epilepsy OR ASD OR autis*).ti,ab,kw.)
2	((‘Fragile X Syndrome’ OR ‘fragile x mental retardation protein’ OR ‘FMR1’ OR ‘Rett Syndrome’ OR ‘Methyl-CpG-Binding Protein 2’ OR MECP2).ti,ab,kw.)
3	((CHD8 OR SCN2A OR SYNGAP1 OR ADNP OR FOXP1 OR POGZ OR ARID1B OR SUV420H1 OR DYRK1A OR SLC6A1 OR GRIN2B OR PTEN OR SHANK3 OR MED13L OR GIGYF1 OR CHD2 OR ANKRD11 OR ANK2 OR ASH1L OR TLK2 OR DNMT3A OR DEAF1 OR CTNNB1 OR KDM6B OR DSCAM OR SETD5 OR KCNQ3 OR SRPR OR KDM5B OR WAC OR SHANK2 OR NRXN1 OR TBL1XR1 OR MYT1L OR BCL11A OR RORB OR RAI1 OR DYNC1H1 OR DPYSL2 OR AP2S1 OR KMT2C OR PAX5 OR MKX OR GABRB3 OR SIN3A OR MBD5 OR MAP1A OR STXBP1 OR CELF4 OR PHF12 OR TBR1 OR PPP2R5D OR TM9SF4 OR PHF21A OR PRR12 OR SKI OR ASXL3 OR SPAST OR SMARCC2 OR TRIP12 OR CREBBP OR TCF4 OR CACNA1E OR GNAI1 OR TCF20 OR FOXP2 OR NSD1 OR TCF7L2 OR LDB1 OR EIF3G OR PHF2 OR KIAA0232 OR VEZF1 OR GFAP OR IRF2BPL OR ZMYND8 OR SATB1 OR RFX3 OR SCN1A OR PPP5C OR TRIM23 OR TRAF7 OR ELAVL3 OR GRIA2 OR LRRC4C OR CACNA2D3 OR NUP155 OR KMT2E OR NR3C2 OR NACC1 OR PTK7 OR PPP1R9B OR GABRB2 OR HDLBP OR TAOK1 OR UBR1 OR TEK OR KCNMA1 OR CORO1A OR HECTD4 OR NCOA1 OR DIP2A).ti, ab,kw.)
4	#1 OR #2 OR #3
	5. (exp animal experiment/ OR exp animal model/ OR exp experimental animal/ OR exp transgenic animal/ OR exp male animal/ OR exp female animal/ OR exp juvenile animal/ OR animal/ OR chordata/ OR vertebrate/ OR tetrapod/ OR exp fish/ OR amniote/ OR exp amphibia/ OR mammal/ OR exp reptile/ OR exp sauropsid/ OR therian/ OR exp monotreme/ OR placental mammal/ OR exp marsupial/ OR Euarchontoglires/ OR exp Afrotheria/ OR exp Boreoeutheria/ OR exp Laurasiatheria/ OR exp Xenarthra/ OR primate/ OR exp Dermoptera/ OR expGlires/ OR exp Scandentia/ OR Haplorhini/ OR exp prosimian/ OR simian/ OR exp tarsiiform/ OR Catarrhini/ OR exp Platyrrhini/ OR ape/ OR exp Cercopithecidae/ OR hominid/ OR exp hylobatidae/ OR exp chimpanzee/ OR exp gorilla/ OR exp orang utan/ OR exp cephalopod/) OR (rat OR rats OR animal OR animals OR mice OR ‘in vivo’ OR mouse OR rabbit OR rabbits OR murine OR pig OR pigs OR dog OR dogs OR bovine OR fish OR vertebrate OR vertebrates OR cat OR cats OR rodent OR rodents OR mammal OR mammals OR chicken OR chickens OR monkey OR monkeys OR sheep OR canine OR canines OR porcine OR cattle OR bird OR birds OR hamster OR hamsters OR primate OR primates OR cow OR cows OR chick OR horse OR horses OR avian OR avians OR calf OR swine OR swines OR xenopus OR turkeys OR bear OR bears OR frog OR frogs OR zebrafish OR goat OR goats OR equine OR calves OR poultry OR macaque OR macaques OR mole OR moles OR ovine OR lamb OR lambs OR fishes OR diptera OR amphibian OR amphibians OR snake OR snakes OR ruminant OR ruminants OR henOR hens OR piglet OR piglets OR feline OR felines OR simian OR simians OR laevis OR trout OR trouts OR teleost OR teleosts OR salmon OR salmons OR seal OR seals OR bull OR bulls OR ewe OR ewes OR hedgehog OR hedgehogs OR macaca OR macacas OR proteus OR pigeon OR pigeons OR bat OR bats OR duck OR ducks OR chimpanzee OR chimpanzees OR baboon OR baboons OR deer OR deers OR rana OR ranas OR carp OR carps OR heifer OR swallow OR swallows OR lizard OR lizards OR canis OR sow OR sows OR cynomolgus OR quail OR quails OR reptile OR reptiles OR turtle OR turtles OR buffalo OR gerbil OR gerbils OR boar OR boars OR squirrel OR squirrels OR oncorhynchus OR mus OR toad OR toads OR fowl OR fowls OR rerio OR danio OR ara OR aras OR musculus OR tadpole OR tadpoles OR mulatta OR
Ovid Embase search strategy	
#	Search terms
	salmo OR ram OR eagle OR eagles OR ferret OR ferrets OR goldfish OR catfish OR whale OR whales OR fox OR foxes OR ape OR apes OR elephant OR elephants OR bos OR marmoset OR marmosets OR cod OR cods OR shark OR sharks OR wolf OR eel OR eels OR auratus OR rattus OR zebra OR zebras OR tilapia OR tilapias OR gilt OR camel OR camels OR squid OR gallus OR marsupial OR marsupials OR vole OR voles OR fascicularis OR ovis OR salmonid OR salmonids OR tiger OR tigers OR dolphin OR dolphins OR robin OR robins OR carpio OR opossum OR opossums OR cyprinus OR salamander OR salamanders OR felis OR mink OR minks OR swan OR swans OR norvegicus OR bufo OR torpedo OR bass OR lamprey OR lampreys OR sus OR python OR pythons OR tetrapod OR tetrapods OR shrew OR shrews OR lionOR lions OR hog OR hogs OR songbird OR songbirds OR oreochromis OR starling OR starlings OR caprine OR carassius OR owl OR owls OR newt OR newts OR papio OR scrofa OR hare OR hares OR gorilla OR gorillas OR flounder OR flounders OR goose OR herring OR herrings OR therian OR buffaloes OR canary OR sparrow OR sparrows OR microtus OR octopus OR troglodytes OR tuna OR amphibia OR chinchilla OR chinchillas OR ide OR oryzias OR cervus OR kangaroo OR kangaroos OR armadillo OR armadillos OR callithrix OR ‘pan troglodytes’ OR saimiri OR cichlid OR cichlids OR donkey OR donkeys OR bream OR char OR chars OR finch OR raccoon OR raccoons OR bothrops OR anguilla OR perch OR cricetus OR seabird OR seabirds OR buck OR bucks OR naja OR coturnix OR salmonids OR geese OR minnow OR minnows OR raptor OR raptors OR merione OR meriones OR rodentia OR elaphus OR amniote OR amniotes OR elasmobranch OR emu OR emus OR peromyscus OR hominid OR hominids OR bubalus OR crotalus OR gull OR gulls OR anas OR anura OR lemur OR lemurs OR crow OR crows OR camelus OR gibbon OR gibbons OR waterfowl OR parrot OR parrots OR eels OR cob OR stickleback OR sticklebacks OR columba OR mesocricetus OR ambystoma OR raven OR ravens OR gadus OR penguin OR penguins OR orangutan OR orangutans OR sturgeon OR sturgeons OR cuniculus OR aves OR virginianus OR cephalopod OR cephalopods OR cebus OR sparus OR tortoise OR tortoises OR guttata OR morhua OR unguiculatus OR dogfish OR vulpes OR mallard OR mallards OR apodemus OR alligator OR alligators OR oryctolagus OR llama OR llamas OR reindeer OR mustela OR duckling OR ducklings OR wolves OR sander OR amazona OR zebu OR badger OR badgers OR dove OR doves OR ictalurus OR capra OR capras OR equus OR camelid OR camelids OR poecilia OR mule OR mules OR perciformes OR salvelinus OR labrax OR cyprinidae OR ariidae OR crocodile OR crocodiles OR fundulus OR dicentrarchus OR clarias OR cercopithecus OR chiroptera OR alpaca OR alpacas OR pike OR pikes OR paralichthys OR puma OR pumas OR didelphis OR pisces OR macropus OR triturusOR bison OR bisons OR epinephelus OR gasterosteus OR panthera OR acipenser OR mackerel OR mackerels OR tamarin OR tamarins OR ostrich OR anolis OR vervet OR vervets OR wallaby OR glareolus OR beaver OR beavers OR dromedary OR catus OR killifish OR pimephales OR promelas OR aotus OR phoca OR panda OR pandas OR porpoise OR porpoises OR myotis OR yak OR yaks OR agkistrodon OR vipera OR otter OR otters OR turbot OR turbots OR squamate OR carnivora OR mullet OR mullets OR hawk OR hawks OR taeniopygia OR seahorse OR seahorses OR ‘poecilia reticulata’ OR falcon OR falcons OR prosimian OR prosimians OR parus OR perca OR fingerling OR fingerlings OR antelope OR antelopes OR tupaia OR passeriformes OR sepia OR saguinus OR coyote OR coyotes OR pongo OR meleagris OR reptilia OR lepus OR psittacine OR hagfish OR warbler OR warblers OR ‘russell s viper’ OR ‘russell s vipers’ OR smolt OR smolts OR budgerigar OR sardine OR sardines OR cavia OR cavias OR hyla OR pleurodeles OR siluriformes OR ‘great tit’ OR ‘great tits’ OR guppy OR bonobo OR bonobos OR rutilus OR trichosurus OR muridae OR phodopus OR channa OR squalus OR lynx OR sturnus OR petromyzon OR vitulina OR monodelphis OR cuttlefish OR adder OR adders OR lepomis OR canaria OR gambusia OR guppies OR xiphophorus OR flatfish OR koala OR koalas OR labeo OR stingray OR stingrays OR chelonia OR lampetra OR spermophilus OR crocodilian OR ‘passer domesticus’ ORsciurus OR artiodactyla OR ranidae OR corvus OR necturus OR platypus OR canaries OR bovid OR lagopus OR trimeresurus OR gariepinus OR marten OR martens OR drosophilidae OR mugil OR sunfish OR porcellus OR cypriniformes OR alouatta OR scophthalmus OR anser OR electrophorus OR putorius OR iguana OR iguanas OR lama OR lamas OR takifugu OR circus OR eptesicus OR flycatcher OR galago OR galagos OR trachemys OR lungfish OR characiformes OR shorebird OR shorebirds OR giraffe OR giraffes OR micropterus OR scyliorhinus OR cichlidae OR loligo OR porcupine OR porcupines OR chub OR chubs OR solea OR pleuronectes OR hylidae OR viperidae OR echis OR sorex OR anchovy OR lagomorph OR ostriches OR vulture OR vultures OR whitefish OR araneus OR jird OR jirds OR tern OR esox OR drake OR drakes OR elapidae OR gallopavo OR chordata OR myodes OR caretta OR serinus OR grouse OR misgurnus OR meles OR blackbird OR blackbirds OR coregonus OR bobwhite OR bobwhites OR heteropneustes OR mammoth OR mammoths OR turdus OR rhinella OR ateles OR characidae OR clupea OR bungarus OR brill OR ‘struthio camelus’ OR sloth OR sloths OR pteropus OR sculpin OR anthropoids OR pollock OR pollocks OR morone OR ‘pan paniscus’ OR litoria OR chipmunk OR chipmunks OR balaenoptera OR marmota OR melopsittacus OR hyrax OR lemming OR lemmings OR halibut OR hylobates OR lates OR caiman OR caimans OR sigmodon OR stenella OR barbel OR barbels OR sterna OR parakeet OR parakeets OR phocoena OR leptodactylus OR canidae OR buteo OR harengus OR gopher OR gophers OR marmot OR marmots OR gosling OR goslings OR platichthys OR gar OR gars OR sebastes OR marsupialia OR notophthalmus OR gazelle OR gazelles OR insectivora OR paridae OR felidae OR russula OR galliformes OR bombina OR colobus OR echidna OR echidnas OR seabass OR syncerus OR plaice OR ‘blue tit’ OR ‘blue tits’ OR pagrus OR catfishes OR cetacea OR barbus OR cygnus OR ficedula OR chamois OR colubridae OR perches OR coelacanth OR fitch OR urodela OR cynops OR martes OR halichoerus OR aix OR salmonidae OR leuciscus OR magpie OR magpies OR silurus OR whiting OR whitings OR anseriformes OR colinus OR rhea OR chlorocebus OR octodon OR acinonyx OR mouflon OR mouflons OR ibex OR tetraodon OR bufonidae OR equidae OR jackal OR cephalopoda OR dendroaspis OR glama OR muskrat OR muskrats OR sable OR sables OR wildebeest OR streptopelia OR albifrons OR vespertilionidae OR woodpecker OR woodpeckers OR muntjac OR muntjacs OR archosaur OR branta OR cricetulus OR megalobrama OR poeciliidae OR desmodus OR snakehead OR snakeheads OR tench OR teal OR teals OR bandicoot OR bandicoots OR apteronotus OR phyllostomidae OR crocidura OR buzzard OR buzzards OR larimichthys OR cercocebus OR pipistrellus OR erithacus OR impala OR impalas OR rousettus OR haddock OR haddocks OR tinca OR ratite OR calidris OR cynoglossus OR hypophthalmichthys OR bullock OR bullocks OR dromedaries OR alectoris OR filly OR salamandra OR cingulata OR bitis OR grus OR ammodytes OR macaw OR macaws OR hypoleuca OR sapajus OR cyprinodontiformes OR hippopotamus OR pelophylax OR capybara OR capybaras OR weasel OR weasels OR cairina OR cynomys OR lutra OR cockatoo OR cockatoos OR lachesis OR lagomorpha OR rupicapra OR daboia OR ‘orang utan’ OR ‘orang utans’ OR platyrrhini OR charadriiformes OR micrurus OR psittaciformes OR spalax OR loris OR mustelidae OR sylvilagus OR vitticeps OR cockatiel OR mustelus OR cottus OR erythrocebus OR dipodomys OR platessa OR callicebus OR loricariidae OR catostomus OR cuneata OR cyanistes OR cyprinodon OR sigmodontinae OR elasmobranchii OR trichechus OR sauropsid OR xenarthra OR dormouse OR perissodactyla OR nautilus OR cirrhinus OR gulo OR gulos OR tragelaphus OR merula OR numida OR sciaenidae OR cerastes OR sciuridae OR gibbosus OR octopuses OR eland OR elands OR phyllomedusa OR pogona OR walrus OR agamidae OR leptodactylidae OR ridibundus OR leontopithecus OR anteater OR anteaters OR pelodiscus OR cebidae OR columbianus OR ‘pelteobagrus fulvidraco’ OR hominoidea OR mandrillus OR ‘zonotrichia leucophrys’ OR agama OR gobiocypris OR ‘bearded dragon’ OR ‘bearded dragons’ OR sarotherodon OR talpa OR discoglossus OR hagfishes OR sphenodon OR gudgeon OR amphiuma OR aythya OR tenrec OR tenrec OR hominidae OR risoria OR salamandridae OR camelidae OR columbiformes OR latimeria OR plover OR plovers OR afrotheria OR ‘falco sparverius’ OR polecat OR polecats OR crotalinae OR salvadora OR tarsier OR lucioperca OR anchovies OR lungfishes OR terrapin OR ‘dromaius novaehollandiae’ OR lateolabrax OR eigenmannia OR pelamis OR theropithecus OR murinae OR gander OR gymnotus OR pseudacris OR gymnophiona OR gymnotiformes OR laticauda OR falconiformes OR dugong OR dugongs OR pintail OR pintails OR rook OR rooks OR lasiurus OR catshark OR catsharks OR micropogonias OR ‘red junglefowl’ OR paddlefish OR ophiophagus OR hollandicus OR nymphicus OR pimelodidae OR aepyceros OR cobitidae OR strigiformes OR cobitis OR dormice OR alytes OR calloselasma OR guanaco OR guanacos OR phasianidae OR ‘round goby’ OR trichogaster OR catarrhini OR eelpout OR eelpouts OR galaxias OR gaur OR pungitius OR suslik OR susliks OR flatfishes OR percidae OR caprinae OR todarodes OR osmerus OR ameiurus OR anthropoidea OR’castor canadensis’ OR pouting OR poutings OR
Ovid Embase search strategy	
#	Search terms
	tetraodontiformes OR arvicolinae OR siamang OR siamangs OR ‘castor fiber’ OR nomascus OR ‘red knot’ OR ‘red knots’ OR syngnathidae OR iguanidae OR eretmochelys OR ursidae OR callimico OR columbidae OR microhylidae OR anaxyrus OR menidia OR pipistrelle OR greylag OR pipidae OR scandentia OR bowfin OR bowfins OR dendrobatidae OR zenaida OR bushbaby OR harrier OR harriers OR macropodidae OR pygerythrus OR clupeidae OR odorrana OR corvidae OR jerboa OR jerboas OR canutus OR hylobatidae OR clupeiformes OR ‘great cormorant’ OR ‘great cormorants’ OR scorpaeniformes OR chondrostean OR garfish OR proboscidea OR psetta OR diapsid OR serotinus OR tetrao OR walruses OR carcharhiniformes OR leucoraja OR pumpkinseed OR dosidicus OR acipenseriformes OR daubentonii OR emberizidae OR gadiformes OR hyraxes OR stizostedion OR wolverine OR wolverines OR lissotriton OR acanthurus OR centrarchidae OR gloydius OR laurasiatheria OR limosa OR psittacula OR leporidae OR proteidae OR zander OR zanders OR arapaima OR bagridae OR cyprinodontidae OR mithun OR pandion OR jackdaw OR jackdaws OR procyonidae OR carus OR jaculus OR salmoniformes OR ‘common sole’ OR ‘common soles’ OR protobothrops OR calamita OR brachyteles OR trionyx OR turdidae ORboidae OR luscinia OR pugnax OR euarchontoglires OR saithe OR saithes OR symphalangus OR aardvark OR aardvarks OR oystercatcher OR oystercatchers OR arius OR corydoras OR poacher OR poachers OR aurochs OR cebuella OR crecca OR lemuridae OR sirenia OR lemmus OR perdix OR glires OR lepidosaur OR muskox OR deinagkistrodon OR pholidota OR holocephali OR cercopithecinae OR clariidae OR agapornis OR doryteuthis OR tyrannidae OR dicroglossidae OR godwit OR godwits OR monedula OR pongidae OR atheriniformes OR colobinae OR lophocebus OR atelidae OR cottidae OR leucopsis OR acanthuridae OR didelphimorphia OR elver OR elvers OR lapponica OR dermoptera OR ‘european hake’ OR ‘european hakes’ OR gerbillinae OR banteng OR hartebeest OR hartebeests OR hogget OR haematopus OR ‘anguis fragilis’ OR ‘grey heron’ OR ‘grey herons’ OR ‘blue whiting’ OR ‘blue whitings’ OR furnariidae OR macrovipera OR esocidae OR lapwing OR lapwings OR mylopharyngodon OR wallabia OR beloniformes OR potoroo OR potoroos OR ‘athene noctua’ OR pleuronectidae OR bushbabies OR muscicapidae OR alligatoridae OR fuligula OR ‘bush baby’ OR guineafowl OR spoonbill OR spoonbills OR viverridae OR catostomidae OR zebrafishes OR ibexes OR vendace OR estrildidae OR monotremata OR sepiella OR ambystomatidae OR shelduck OR shelducks OR treeshrew OR treeshrews OR hoplobatrachus OR pochard OR hoolock OR hoolocks OR lynxes OR antilope OR antilopes OR blackbuck OR blackbucks OR cricetinae OR paramisgurnus OR skylark OR skylarks OR soleidae OR allobates OR ‘northern wheatear’ OR ‘northern wheatears’ OR pitheciidae OR takin OR theria OR vanellus OR galaxiidae OR lorisidae OR ostralegus OR palaeognathae OR ‘stone loach’ OR alauda OR callitrichinae OR caniformia OR duttaphrynus OR ictaluridae OR osteoglossiformes OR poultries OR curema OR ‘ruddy turnstone’ OR ‘ruddy turnstones’ OR sheatfish OR sunfishes OR centropomidae OR hemachatus OR platalea OR thamnophilidae OR ‘song thrush’ OR atherinopsidae OR siluridae OR tadorna OR chroicocephalus OR ermine OR ermines OR gavialis OR ruff OR tupaiidae OR diprotodontia OR hyaenidae OR antilopinae OR crocodylidae OR herpestidae OR hippopotamidae OR ‘northern shoveler’ OR ‘round gobies’ OR cheirogaleidae OR indriidae OR fundulidae OR pythonidae OR rhynchocephalia OR anodorhynchus OR ‘red-backed shrike’ OR ‘red-backed shrikes’ OR triakidae OR phalangeridae OR aoudad OR boreoeutheria OR ‘eurasian jay’ OR ‘eurasian jays’ OR feliformia OR haplorhini OR osteoglossidae OR paenungulata OR struthioniformes OR ferina OR sanderling OR sanderlings OR spheniscidae OR cuttlefishes OR cygnet OR dasycneme OR gadwall OR gadwalls OR ‘pelobates fuscus’ OR wryneck OR wrynecks OR afrosoricida OR culaea OR ‘dover sole’ OR ‘dover soles’ OR paralichthyidae OR passeridae OR osteolaemus OR ‘song thrushes’ OR bluethroat OR bluethroats OR hydrophiidae OR megrim OR mephitidae OR strepsirhini OR tomistoma OR epidalea OR osmeriformes OR ‘bush babies’ OR tarsiiform OR atelinae OR bufotes OR ‘eurasian coot’ OR ‘eurasian coots’ OR galagidae OR geopelia OR philomachus OR tubulidentata OR bombinatoridae OR pelobatidae OR tachysurus OR ailuridae OR woodlark OR woodlarks OR alcelaphinae OR redshank OR redshanks OR salientia OR ‘sand smelt’ OR ‘sand smelts’ OR woodmice OR woodmouse OR dasyproctidae OR ‘eurasian wigeon’ OR ‘eurasianwigeons’ OR garganey OR garganeys OR ‘lemon sole’ OR ‘lemon soles’ OR ‘common dab’ OR ‘common dabs’ OR graylag OR graylags OR leucorodia OR osphronemidae OR bewickii OR ‘common moorhen’ OR ‘common moorhens’ OR decapodiformes OR gobbler OR gobblers OR odontophoridae OR paddlefishes OR eutheria OR salmonine OR esociformes OR ‘eurasian woodcock’ OR ‘eurasian woodcocks’ OR ‘european smelt’ OR ‘european smelts’ OR goldfishes OR tenches OR tyranni OR ‘common chaffinch’ OR ‘common chaffinchs’ OR ‘common redstart’OR ‘common redstarts’ OR ‘common roach’ OR ‘common roachs’ OR ‘great knot’ OR ‘great knots’ OR potoroidae OR alytidae OR coregonine OR dipteral OR leveret OR ‘poeciliopsis gracilis’ OR amphiumidae OR batrachoidiformes OR ‘bighead goby’ OR heteropneustidaeOR lullula OR ‘norway pout’ OR ‘norway pouts’ OR sipunculida OR dogfishes OR sebastidae OR tarsiidae OR alethinophidia OR ‘common nase’ OR ‘common nases’ OR ‘common sandpiper’ OR ‘common sandpipers’ OR ‘eurasian blackcap’ OR ‘eurasian blackcaps’ OR pterocnemia OR syngnathiformes OR ‘common chaffinches’ OR eupleridae OR octopodiformes OR phascolarctidae OR scophthalmidae OR ‘starry smooth-hound’ OR ‘starry smooth-hounds’ OR whitefishes OR cuniculidae OR ‘european sprat’ OR ‘european sprats’ OR ‘rosy bitterling’ OR ‘rosy bitterlings’ OR ‘common dace’ OR ‘common daces’ OR ‘lesser weever’ OR ‘lesser weevers’ OR scaldfish OR ‘water rail’ OR ‘water rails’ OR alouattinae OR centrarchiformes OR ‘common whitethroat’ OR ‘common whitethroats’ OR gavialidae OR ‘grey gurnard’ OR ‘grey gurnards’ OR lateolabracidae OR rheiformes OR ‘tub gurnard’ OR ‘tub gurnards’ OR ‘common chiffchaff’ OR ‘common chiffchaffs’ OR garfishes OR ‘lesser whitethroat’ OR ‘lesser whitethroats’ OR myoxidae OR seabasses OR spariformes OR umbridae OR ‘yellow boxfish’ OR anabantiformes OR aotidae OR ‘common bleak’ OR ‘common bleaks’ OR ‘common rudd’ OR ‘common rudds’ OR ‘greater pipefish’ OR hapale OR nandiniidae OR ‘stone loaches’ OR whinchat OR whinchats OR acanthuriformes OR ‘brotula barbata’ OR ‘common ling’ OR ‘common lings’ OR ‘common roaches’ OR cottonrat OR cottonrats OR douroucoulis OR dromaiidae OR fitches OR fitchew OR galaxiiformes OR laprine OR saimiriinae OR solenette OR tarsii OR ‘tompot blenny’ OR ‘common dragonet’ OR ‘common dragonets’OR ‘longspined bullhead’ OR ‘longspined bullheads’ OR monotremate OR monotremates OR pempheriformes OR perdicinae OR presbytini OR smegmamorpha OR ‘bighead gobies’ OR ‘carangaria incertae sedis’ OR coiidae OR ‘fivebeard rockling’ OR foulmart OR foumart ORgrasskeet OR ‘greater pipefishes’ OR ibices OR millionfish OR muguliformes OR ‘norwegian topknot’ OR peewit OR ‘red sea sailfin tang’ OR rupicapras OR sheatfishes OR ‘tompot blennies’ OR ‘twait shad’ OR ‘yellow boxfishes’).ti, ab,kw.
6	#4 AND #5
7	((‘systematic review’ OR ‘meta-analysis’ OR metaanalysis).ti. OR (((meta-analyses OR meta-analysis OR metaanalyses OR metaanalysis OR ‘systematic overview’).ti, ab,de. OR ‘systematic reviews’.jt. OR ‘meta analysis’.jt. OR ‘Meta synthesis’.ti, ab,de. OR (Systematic* adj2 (Review OR literature OR Reviews OR survey OR search*)).ti,ab,de.) and (‘Data collection’ OR ‘Data extraction’ OR ‘Inclusion Criteria’ OR ‘Exclusion criteria’ OR Search* OR Literature OR Pubmed OR Medline OR Embase OR selection OR Web of Science OR Google OR Scopus OR BIOSIS).ti,ab,de.))
8	#6 AND #7
Web of Science search strategy	
#	Search terms
1	(TS = (neurodevelop* OR neurodevelop* delay* OR intellectual disabilit* OR epilepsy OR ASD OR autis*))
2	(TS = (‘Fragile X Syndrome’ OR ‘fragile x mental retardation protein’ OR ‘FMR1’ OR ‘Rett Syndrome’ OR ‘Methyl-CpG-Binding Protein 2’ OR MECP2))
3	(TS = (CHD8 OR SCN2A OR SYNGAP1 OR ADNP OR FOXP1 OR POGZ OR ARID1B OR SUV420H1 OR DYRK1A OR SLC6A1 OR GRIN2B OR PTEN OR SHANK3 OR MED13L OR GIGYF1 OR CHD2 OR ANKRD11 OR ANK2 OR ASH1L OR TLK2 OR DNMT3A OR DEAF1 OR CTNNB1 OR KDM6B OR DSCAM OR SETD5 OR KCNQ3 OR SRPR OR KDM5B OR WAC OR SHANK2 OR NRXN1 OR TBL1XR1 OR MYT1L OR BCL11A OR RORB OR RAI1 OR DYNC1H1 OR DPYSL2 OR AP2S1 OR KMT2C OR PAX5 OR MKX OR GABRB3 OR SIN3A OR MBD5 OR MAP1A OR STXBP1 OR CELF4 OR PHF12 OR TBR1 OR PPP2R5D OR TM9SF4 OR PHF21A OR PRR12 OR SKI OR ASXL3 OR SPAST OR SMARCC2 OR TRIP12 OR CREBBP OR TCF4 OR CACNA1E OR GNAI1 OR TCF20 OR FOXP2 OR NSD1 OR TCF7L2 OR LDB1 OR EIF3G OR PHF2 OR KIAA0232 OR VEZF1 OR GFAP OR IRF2BPL OR ZMYND8 OR SATB1 OR RFX3 OR SCN1A OR PPP5C OR TRIM23 OR TRAF7 OR ELAVL3 OR GRIA2 OR LRRC4C OR CACNA2D3 OR NUP155 OR KMT2E OR NR3C2 OR NACC1 OR PTK7 OR PPP1R9B OR GABRB2 OR HDLBP OR TAOK1 OR UBR1 OR TEK OR KCNMA1 OR CORO1A OR HECTD4 OR NCOA1 OR DIP2A))
4	#1 OR #2 OR #3
5	TS = (rat OR rats OR animal OR animals OR mice OR ‘in vivo’ OR mouse OR rabbit OR rabbits OR murine OR pig OR pigs OR dog OR dogs OR bovine OR fish OR vertebrate OR vertebrates OR cat OR cats OR rodent OR rodents OR mammal OR mammals OR chicken OR chickens OR monkey OR monkeys OR sheep OR canine OR canines OR porcine OR cattle OR bird OR birds OR hamster OR hamsters OR primate OR primates OR cow OR cows OR chick OR horse OR horses OR avian OR avians OR calf OR swine OR swines OR xenopus OR turkeys OR bear OR bears OR frog OR frogs OR zebrafish OR goat OR goats OR equine OR calves OR poultry OR macaque OR macaques OR mole OR moles OR ovine OR lamb OR lambs OR fishes OR diptera OR amphibian OR amphibians OR snake OR snakes OR ruminant OR ruminants OR hen OR hens OR piglet OR piglets OR feline OR felines OR simian OR simians OR laevis OR trout OR trouts OR teleost OR teleosts OR salmon OR salmons OR seal OR seals OR bull OR bulls OR ewe OR ewes OR hedgehog OR hedgehogs OR macaca OR macacas OR proteus OR pigeon ORpigeons OR bat OR bats OR duck OR ducks OR chimpanzee OR chimpanzees OR baboon OR baboons OR deer OR rana OR ranas OR carp OR carps OR heifer OR swallow OR swallows OR lizard OR lizards OR canis OR sow OR sows OR cynomolgus OR quail OR quails OR reptile OR reptiles OR turtle OR turtles OR buffalo OR gerbil OR gerbils OR boar OR boars OR squirrel OR squirrels OR oncorhynchus OR mus OR toad OR toads OR fowl OR fowls OR rerio OR danio OR ara OR aras OR musculus OR tadpole OR tadpoles OR mulatta OR salmo OR ram OR eagle OR eagles OR ferret OR ferrets OR goldfish OR catfish OR whale OR whales OR fox OR foxes OR ape OR apes OR elephant OR elephants OR bos OR marmoset OR marmosets OR cod OR cods OR shark OR sharks OR wolf OR eel OR eels OR auratus OR rattus OR zebra OR zebras OR tilapia OR tilapias OR gilt OR camel OR camels OR squid OR gallus OR marsupial OR marsupials OR vole OR voles OR fascicularis OR ovis OR salmonid OR salmonids OR tiger OR tigers OR dolphin OR dolphins OR robin OR robins OR carpio OR opossumOR opossums OR cyprinus OR salamander OR salamanders OR felis OR mink OR minks OR swan OR swans OR norvegicus OR bufo OR torpedo OR bass OR lamprey OR lampreys OR sus OR python OR pythons OR tetrapod OR tetrapods OR shrew OR shrews OR lion OR lions OR hogOR hogs OR songbird OR songbirds OR oreochromis OR starling OR starlings OR caprine OR carassius OR owl OR owls OR newt OR newts OR papio OR scrofa OR hare OR hares OR gorilla OR gorillas OR flounder OR flounders OR goose OR herring OR herrings OR therianOR buffaloes OR canary OR sparrow OR sparrows OR microtus OR octopus OR troglodytes OR tuna OR amphibia OR chinchilla OR chinchillas OR ide OR oryzias OR cervus OR kangaroo OR kangaroos OR armadillo OR armadillos OR callithrix OR ‘pan troglodytes’ OR saimiri OR cichlid OR cichlids OR donkey OR donkeys OR bream OR char OR chars OR finch OR raccoon OR raccoons OR bothrops OR anguilla OR perch OR cricetus OR seabird OR seabirds OR buck OR bucks OR naja OR coturnix OR salmonids OR geese OR minnow OR minnows ORraptor OR raptors OR merione OR meriones OR rodentia OR elaphus OR amniote OR amniotes OR elasmobranch OR emu OR emus OR peromyscus OR hominid OR hominids OR bubalus OR crotalus OR gull OR gulls OR anas OR anura OR lemur OR lemurs OR crow OR crows OR camelus OR gibbon OR gibbons OR waterfowl OR parrot OR parrots OR eels OR cob OR stickleback OR sticklebacks OR columba OR mesocricetus OR ambystoma OR raven OR ravens OR gadus OR penguin OR penguins OR orangutan OR orangutans OR sturgeon OR sturgeons OR cuniculus OR aves OR virginianus OR cephalopod OR cephalopods OR cebus OR sparus OR tortoise OR tortoises OR guttata OR morhua OR unguiculatus OR dogfish OR vulpes OR mallard OR mallards OR apodemus OR alligator OR alligators OR oryctolagus OR llama OR llamas OR reindeer OR mustela OR duckling OR ducklings OR wolves OR sander OR amazona OR zebu OR badger OR badgers OR dove OR doves OR ictalurus OR capra OR capras OR equus OR camelid OR camelids OR poecilia OR mule OR mules OR perciformes OR salvelinus OR labrax OR cyprinidae OR ariidae OR crocodile OR crocodiles OR fundulus OR dicentrarchus OR clarias OR cercopithecus OR chiroptera OR alpaca OR alpacas OR pike OR pikes OR paralichthys OR puma OR pumas OR didelphis OR pisces OR macropus OR triturus OR bison OR bisons OR epinephelus OR gasterosteus OR panthera OR acipenser OR mackerel OR mackerels OR tamarin OR tamarins OR ostrich OR anolis OR vervet OR vervets OR wallaby OR glareolus OR beaver OR beavers OR dromedary OR catus OR killifish OR pimephales OR promelas OR aotus OR phoca OR panda OR pandas OR porpoise OR porpoises OR myotis OR yak OR yaks OR agkistrodon OR vipera OR otter OR otters OR turbot OR turbots OR squamate OR carnivora OR mullet OR mullets OR hawk OR hawks OR taeniopygia OR seahorse OR seahorses OR ‘poecilia reticulata’ OR falcon OR falcons OR prosimian OR prosimians OR parus OR perca OR fingerling OR fingerlings OR antelope OR antelopes OR tupaia OR passeriformes OR sepia OR saguinus OR coyote OR coyotes OR pongo OR meleagris OR reptilia OR lepus OR psittacine OR hagfish OR warbler OR warblers OR ‘russell s viper’ OR ‘russell s vipers’ OR smolt OR smolts OR budgerigar OR sardine OR sardines OR cavia OR cavias OR hyla OR pleurodeles OR siluriformes OR ‘great tit’ OR ‘great tits’ OR guppy OR bonobo OR bonobos OR rutilus OR trichosurus OR muridae OR phodopus OR channa OR squalus OR lynx OR sturnus OR petromyzon OR vitulina OR monodelphis OR cuttlefish OR adder OR adders OR lepomis OR canaria OR gambusia OR guppies OR xiphophorus OR flatfish OR koala ORkoalas OR labeo OR stingray OR stingrays OR chelonia OR lampetra OR spermophilus OR crocodilian OR ‘passer domesticus’ OR sciurus OR artiodactyla OR ranidae OR corvus OR necturus OR platypus OR canaries OR bovid OR lagopus OR trimeresurus OR gariepinus ORmarten OR martens OR drosophilidae OR mugil OR sunfish OR porcellus OR cypriniformes OR alouatta OR scophthalmus OR anser OR electrophorus OR putorius OR iguana OR iguanas OR lama OR lamas OR takifugu OR circus OR eptesicus OR flycatcher OR galago OR galagos OR trachemys OR lungfish OR characiformes OR shorebird OR shorebirds OR giraffe OR giraffes OR micropterus OR scyliorhinus OR cichlidae OR loligo OR porcupine OR porcupines OR chub OR chubs OR solea OR pleuronectes OR hylidae OR viperidae OR echis OR sorex OR anchovy OR lagomorph OR ostriches OR vulture OR vultures OR whitefish OR araneus OR jird OR jirds OR tern OR esox OR drake OR drakes OR elapidae OR gallopavo OR chordata OR myodes OR caretta OR serinus OR grouse OR misgurnus OR meles OR blackbird OR blackbirds OR coregonus OR bobwhite OR bobwhites OR heteropneustes OR mammoth OR mammoths OR turdus OR rhinella OR ateles OR characidae OR clupea OR bungarus OR brill OR ‘struthio camelus’ OR sloth OR sloths OR pteropus OR sculpin OR anthropoids OR pollock OR pollocks OR morone OR ‘pan paniscus’ OR litoria OR chipmunk OR
Web of Science search strategy	
#	Search terms
	chip munks OR balaenoptera OR marmota OR melopsittacus OR hyrax OR lemming OR lemmings OR halibut OR hylobates OR lates OR caiman OR caimans OR sigmodon OR stenella OR barbel OR barbels ORsterna OR parakeet OR parakeets OR phocoena OR leptodactylus OR canidae OR buteo OR harengus OR gopher OR gophers OR marmot OR marmots OR gosling OR goslings OR platichthys OR gar OR gars OR sebastes OR marsupialia OR notophthalmus OR gazelle OR gazelles OR insectivora OR paridae OR felidae OR russula OR galliformes OR bombina OR colobus OR echidna OR echidnas OR seabass OR syncerus OR plaice OR ‘blue tit’ OR ‘blue tits’ OR pagrus OR catfishes OR cetacea OR barbus OR cygnus OR ficedula OR chamois OR colubridae OR perches OR coelacanth OR fitch OR urodela OR cynops OR martes OR halichoerus OR aix OR salmonidae OR leuciscus OR magpie OR magpies OR silurus OR whiting OR whitings OR anseriformes OR colinus OR rhea OR chlorocebus OR octodon OR acinonyx OR mouflon OR mouflons OR ibex OR tetraodon OR bufonidae OR equidae OR jackal OR cephalopoda OR dendroaspis OR glama OR muskrat OR muskrats OR sable OR sables OR wildebeest OR streptopelia OR albifrons OR vespertilionidae OR woodpecker OR woodpeckers OR muntjac OR muntjacs OR archosaur OR branta OR cricetulus OR megalobrama OR poeciliidae OR desmodus OR snakehead OR snakeheads OR tench OR teal OR teals OR bandicoot OR bandicoots OR apteronotus OR phyllostomidae OR crocidura OR buzzard OR buzzards OR larimichthys OR cercocebus OR pipistrellus OR erithacus OR impala OR impalas OR rousettus OR haddock OR haddocks OR tinca OR ratite OR calidris OR cynoglossus OR hypophthalmichthys OR bullock OR bullocks OR dromedaries OR alectoris OR filly OR salamandra OR cingulata OR bitis OR grus OR ammodytes OR macaw OR macaws OR hypoleuca OR sapajus OR cyprinodontiformes OR hippopotamus OR pelophylax OR capybara OR capybaras OR weasel OR weasels OR cairina OR cynomys OR lutra OR cockatoo OR cockatoos OR lachesis OR lagomorpha OR rupicapra OR daboia OR ‘orang utan’ OR ‘orang utans’ OR platyrrhini OR charadriiformes OR micrurus OR psittaciformes OR spalax OR loris OR mustelidae OR sylvilagus OR vitticeps OR cockatiel OR mustelus OR cottus OR erythrocebus OR dipodomys OR platessa OR callicebus OR loricariidae OR catostomus OR cuneata OR cyanistes OR cyprinodon OR sigmodontinae OR elasmobranchii OR trichechus OR sauropsid OR xenarthra OR dormouse OR perissodactyla OR nautilus OR cirrhinus OR gulo OR gulos OR tragelaphus OR merula OR numidaOR sciaenidae OR cerastes OR sciuridae OR gibbosus OR octopuses OR eland OR elands OR phyllomedusa OR pogona OR walrus OR agamidae OR leptodactylidae OR ridibundus OR leontopithecus OR anteater OR anteaters OR pelodiscus OR cebidae OR columbianus OR ‘pelteobagrus fulvidraco’ OR hominoidea OR mandrillus OR ‘zonotrichia leucophrys’ OR agama OR gobiocypris OR ‘bearded dragon’ OR ‘bearded dragons’ OR sarotherodon OR talpa OR discoglossus OR hagfishes OR sphenodon OR gudgeon OR amphiuma OR aythya OR tenrec OR tenrec OR hominidae OR risoria OR salamandridae OR camelidae OR columbiformes OR latimeria OR plover OR plovers OR afrotheria OR ‘falco sparverius’ OR polecat OR polecats OR crotalinae OR salvadora OR tarsier OR lucioperca OR anchovies OR lungfishes OR terrapin OR ‘dromaius novaehollandiae’ OR lateolabrax OR eigenmannia OR pelamis OR theropithecus OR murinae OR gander OR gymnotus OR pseudacris OR gymnophiona OR gymnotiformes OR laticauda OR falconiformes OR dugong OR dugongs OR pintail OR pintails OR rook ORrooks OR lasiurus OR catshark OR catsharks OR micropogonias OR ‘red junglefowl’ OR paddlefish OR eutheria OR ophiophagus OR hollandicus OR nymphicus OR pimelodidae OR aepyceros OR cobitidae OR strigiformes OR cobitis OR dormice OR alytes OR calloselasma OR guanaco OR guanacos OR phasianidae OR ‘round goby’ OR trichogaster OR catarrhini OR eelpout OR eelpouts OR galaxias OR gaur OR pungitius OR suslik OR susliks OR flatfishes OR percidae OR caprinae OR todarodes OR osmerus OR ameiurus OR anthropoidea OR ‘castor canadensis’ OR pouting OR poutings OR tetraodontiformes OR arvicolinae OR siamang OR siamangs OR ‘castor fiber’ OR nomascus OR ‘red knot’ OR ‘red knots’ OR syngnathidae OR iguanidae OR eretmochelys OR ursidae OR callimico OR columbidae OR microhylidaeOR anaxyrus OR menidia OR pipistrelle OR greylag OR pipidae OR scandentia OR bowfin OR bowfins OR dendrobatidae OR zenaida OR bushbaby OR harrier OR harriers OR macropodidae OR pygerythrus OR clupeidae OR odorrana OR corvidae OR jerboa OR jerboas OR canutus OR hylobatidae OR clupeiformes OR ‘great cormorant’ OR ‘great cormorants’ OR scorpaeniformes OR chondrostean OR garfish OR proboscidea OR psetta OR diapsid OR serotinus OR tetrao OR walruses OR carcharhiniformes OR leucoraja OR pumpkinseed OR dosidicus OR acipenseriformes OR daubentonii OR emberizidae OR gadiformes OR hyraxes OR stizostedion OR wolverine OR wolverines OR lissotriton OR acanthurus OR centrarchidae OR gloydius OR laurasiatheria OR limosa OR psittacula OR leporidae OR proteidae OR zander ORzanders OR arapaima OR bagridae OR cyprinodontidae OR mithun OR pandion OR jackdaw OR jackdaws OR procyonidae OR carus OR jaculus OR salmoniformes OR ‘common sole’ OR ‘common soles’ OR protobothrops OR calamita OR brachyteles OR trionyx OR turdidae OR boidae OR luscinia OR pugnax OR euarchontoglires OR saithe OR saithes OR symphalangus OR aardvark OR aardvarks OR oystercatcher OR oystercatchers OR arius OR corydoras OR poacher OR poachers OR aurochs OR cebuella OR crecca OR lemuridae OR sirenia OR lemmus OR perdix OR glires OR lepidosaur OR muskox OR deinagkistrodon OR pholidota OR holocephali OR cercopithecinae OR clariidae OR agapornis OR doryteuthis OR tyrannidae OR dicroglossidae OR godwit OR godwits OR monedula OR pongidae OR atheriniformes OR colobinae OR lophocebus OR atelidae OR cottidae OR leucopsis OR acanthuridae OR didelphimorphia OR elver OR elvers OR lapponica OR dermoptera OR ‘european hake’ OR ‘european hakes’ OR gerbillinae OR banteng OR hartebeest OR hartebeests OR hogget OR haematopus OR ‘anguis fragilis’ OR ‘grey heron’ OR ‘grey herons’ OR ‘blue whiting’ OR ‘blue whitings’ OR furnariidae OR macrovipera OR esocidae OR lapwing OR lapwings OR mylopharyngodon OR wallabia OR beloniformes OR potoroo OR potoroos OR ‘athene noctua’ OR pleuronectidae OR bushbabies OR muscicapidae OR alligatoridae OR fuligula OR ‘bush baby’ OR guineafowl OR spoonbill OR spoonbills OR viverridae OR catostomidae OR zebrafishes OR ibexes OR vendace OR estrildidae OR monotremata OR sepiella OR ambystomatidae OR shelduck OR shelducks OR treeshrew OR treeshrews OR hoplobatrachus OR pochard OR hoolock OR hoolocks OR lynxes OR antilope OR antilopes OR blackbuck OR blackbucks OR cricetinae OR paramisgurnus OR skylark OR skylarks OR soleidae OR allobates OR ‘northern wheatear’ OR ‘northern wheatears’ OR pitheciidae OR takin OR theria OR vanellus OR galaxiidae OR lorisidae OR ostralegus OR palaeognathae OR ‘stone loach’ OR alauda OR callitrichinae OR caniformia OR duttaphrynus OR ictaluridae OR osteoglossiformes OR poultries OR curema OR ‘ruddy turnstone’ OR ‘ruddy turnstones’ OR sheatfish OR sunfishes OR centropomidae OR hemachatus OR platalea OR thamnophilidae OR ‘song thrush’ OR atherinopsidae OR siluridae OR tadorna OR chroicocephalus OR ermine OR ermines OR gavialis OR ruffe OR tupaiidae OR diprotodontia OR hyaenidae OR antilopinae OR crocodylidae OR herpestidae OR hippopotamidae OR ‘northern shoveler’ OR ‘round gobies’ OR cheirogaleidae OR indriidae OR fundulidae OR pythonidae OR rhynchocephalia OR anodorhynchus OR ‘red-backed shrike’ OR ‘red-backed shrikes’ OR triakidae OR phalangeridae OR aoudad OR boreoeutheria OR ‘eurasian jay’ OR ‘eurasian jays’ OR feliformia OR haplorhini OR osteoglossidae OR paenungulata OR struthioniformes OR ferina OR sanderling OR sanderlings OR spheniscidae OR cuttlefishes OR cygnet OR dasycneme OR gadwall OR gadwalls OR ‘pelobates fuscus’ OR wryneck OR wrynecks OR afrosoricida OR culaea OR ‘dover sole’ OR ‘dover soles’ OR paralichthyidae OR passeridae OR osteolaemus OR ‘song thrushes’ OR bluethroat OR bluethroats OR hydrophiidae OR megrim OR mephitidae OR strepsirhini OR tomistoma OR epidalea OR osmeriformes OR ‘bush babies’ OR tarsiiform OR atelinae OR bufotes OR ‘eurasian coot’ OR ‘eurasian coots’ OR galagidae OR geopelia OR philomachus OR tubulidentata OR bombinatoridae OR pelobatidae OR tachysurus OR ailuridae OR woodlark OR woodlarks OR alcelaphinae OR redshank OR redshanks OR salientia OR ‘sand smelt’ OR ‘sand smelts’ OR woodmice OR woodmouse OR dasyproctidae OR ‘eurasian wigeon’
Web of Science search strategy	
#	Search terms
	OR ‘eurasian wigeons’ OR garganey OR garganeys OR ‘lemon sole’ OR ‘lemon soles’ OR ‘common dab’ OR ‘common dabs’ OR graylag OR graylags OR leucorodia OR osphronemidae OR bewickii OR ‘common moorhen’ OR ‘common moorhens’ OR decapodiformes OR gobbler OR gobblers OR odontophoridae OR paddlefishes OR salmonine OR esociformes OR ‘eurasian woodcock’ OR ‘eurasian woodcocks’ OR ‘european smelt’ OR ‘european smelts’ OR goldfishes OR tenches OR tyranni OR ‘common chaffinch’ OR ‘common chaffinchs’ OR ‘common redstart’ OR ‘common redstarts’ OR ‘common roach’ OR ‘common roachs’ OR ‘great knot’ OR ‘great knots’ OR potoroidae OR alytidae OR coregonine OR dipteral OR leveret OR ‘poeciliopsis gracilis’ OR amphiumidae OR batrachoidiformes OR ‘bighead goby’ OR heteropneustidae OR lullula OR ‘norway pout’ OR ‘norway pouts’ OR sipunculida OR dogfishes OR sebastidae OR tarsiidae OR alethinophidia OR ‘common nase’ OR ‘common nases’ OR ‘common sandpiper’ OR ‘common sandpipers’ OR ‘eurasian blackcap’ OR ‘eurasian blackcaps’ OR pterocnemia OR syngnathiformes OR ‘common chaffinches’ OR eupleridae OR octopodiformes OR phascolarctidae OR scophthalmidae OR ‘starry smooth-hound’ OR ‘starry smooth-hounds’ OR whitefishes OR cuniculidae OR ‘european sprat’ OR ‘european sprats’ OR ‘rosy bitterling’ OR ‘rosy bitterlings’ OR ‘common dace’ OR ‘common daces’ OR ‘lesser weever’ OR ‘lesser weevers’ OR scaldfish OR ‘water rail’ OR ‘water rails’ OR alouattinae OR centrarchiformes OR ‘common whitethroat’ OR ‘common whitethroats’ OR gavialidae OR ‘grey gurnard’ OR ‘greygurnards’ OR lateolabracidae OR rheiformes OR ‘tub gurnard’ OR ‘tub gurnards’ OR ‘common chiffchaff’ OR ‘common chiffchaffs’ OR garfishes OR ‘lesser whitethroat’ OR ‘lesser whitethroats’ OR myoxidae OR seabasses OR spariformes OR umbridae OR ‘yellow boxfish’ OR anabantiformes OR aotidae OR ‘common bleak’ OR ‘common bleaks’ OR ‘common rudd’ OR ‘common rudds’ OR ‘greater pipefish’ OR hapale OR nandiniidae OR ‘stone loaches’ OR whinchat OR whinchats OR acanthuriformes OR ‘brotula barbata’ OR ‘common ling’ OR ‘common lings’ OR ‘common roaches’ OR cottonrat OR cottonrats OR douroucoulis OR dromaiidae OR fitches OR fitchew OR galaxiiformes OR laprine OR saimiriinae OR solenette OR tarsii OR ‘tompot blenny’ OR ‘common dragonet’ OR ‘common dragonets’ OR ‘longspinedbullhead’ OR ‘longspined bullheads’ OR monotremate OR monotremates OR pempheriformes OR perdicinae OR presbytini OR smegmamorpha OR ‘bighead gobies’ OR ‘carangaria incertae sedis’ OR coiidae OR ‘fivebeard rockling’ OR foulmart OR foumart OR grasskeet OR ‘greater pipefishes’ OR ibices OR millionfish OR muguliformes OR ‘norwegian topknot’ OR peewit OR ‘red sea sailfin tang’ OR rupicapras OR sheatfishes OR ‘tompot blennies’ OR ‘twait shad’ OR ‘yellow boxfishes’)
6	#5 AND #6
7	(TS = ((‘systematic review’ OR ‘systematic reviews’ OR ‘meta-analyses’ OR ‘meta-analysis’ OR ‘metaanalyses’ OR ‘metaanalysis’ OR ‘systematic literature review’ OR ‘Systematic survey’[tiab] OR ‘systematic overview’ OR ‘Systematically review’ OR ‘Systematically searched’ OR ‘Systematic search’ OR ‘Meta synthesis’ OR ‘literature search’ OR ‘literature searches’ OR ‘literature searching’ OR ‘data collection’ OR ‘electronic-database*’ OR ‘databases-search*’ OR ‘electronic-search*’ OR ‘comprehensive-search*’ OR ‘literature search’ OR ‘literature searches’ OR ‘literature searching’ OR ‘data collection’) AND (Pubmed OR Medline OR Embase OR selection OR Web of Science OR Google OR Scopus OR BIOSIS)))
7	#6 AND #7

## Appendix 2

### Checklist to assess the state of reporting within preclinical systematic reviews

**Table:** This checklist is taken from Hunniford et al. (2021).

Section	#	Item
**Title**	1	Identify the report as systematic review in title
	2	Identify that the report contains animal data in title (preclinical, *in vivo* or synonym)
**Intro**	3	Describe the human condition being modelled (e.g. describe what is already known)
	4	Describe the biological rationale for testing the intervention (e.g. how would the intervention affect the condition)
	5	Provide an explicit statement of the question(s) the review addresses (specify the main objectives of the review, ideally in PICO format)
**Methods**	6	Indicate whether a review protocol was registered a priori
	a	Where can the protocol be accessed and indicate the name of the protocol registry OR state that it is not available
	b	Indicate any deviations from the protocol OR that there were no deviations
	7	Eligibility criteria: Describe the animal species to be included in the review (e.g. only mice, vertebrates, large animals)
	8	Eligibility criteria: Describe the animal model to be included in the review (methods of disease induction, age, sex, etc.)
	9	Eligibility criteria: Describe the intervention/exposure of interest
	10	Eligibility criteria: Describe the comparators and/or control population
	11	Eligibility criteria: Describe the primary outcomes of interest (what is being measured/assessed in primary studies)
	12	Eligibility criteria: Describe the timing (prevention vs rescue) of intervention, IF applicable
	13	Indicate where a full search strategy of all data bases OR representative search strategy can be accessed
	14	Describe inclusion limits (years conducted, language, AND publication type)
	15	Describe the study screening/selection process
	a	Report the platform used to screen and select studies (Excel, Access, DistillerSR, SyRF)
	16	State the number of independent screeners
	17	Describe methods for extracting numerical data from reports (e.g. data in bar graph, or non-text presentation), IF applicable^a^
	a	Report the platform and tools used to extract numerical data (Graph2data, Engauge)
	18	Report number of independent reviewers extracting data
	19	Describe methods and tool used to measure study quality/risk of bias in individual studies (e.g. SYRCLE tool, CAMARADES tool)
	20	Describe methods to assess construct validity in individual studies
	21	Describe methods for assessing publication bias of included studies, IF applicable
	22	Describe methods for synthesising the quantitative effect measures of included studies (e.g. risk ratio, mean difference), IF applicable^a^
	23	Describe methods for any data transformation needed to make extracted data suitable for analysis (e.g. only sample size range), IF applicable^a^
	24	Describe methods for handling shared control groups (common issue in analysis of preclinical studies), IF applicable^a^
	25	Describe methods for assessing heterogeneity between individual studies, IF applicable^a^
	26	Describe methods for handling effect sizes over multiple time points (e.g. used all time points or latest time point), IF applicable^a^
	27	Describe methods for sub-group and sensitivity analysis, IF applicable^a^
**Results**	28	Report the number of included reports (individual references/publication) included in the review
	a	Provides a list or table of individual studies with data or references
	29	Report the number of eligible experiments included in the analysis (eligible animal experiments in individual reports)
	30	Include a PRISMA flow diagram (or equivalent) of study selection process
	31	Study characteristics: Report animal species
	32	Study characteristics: Report animal model details (e.g. method of disease induction, age, sex)
	33	Study characteristics: Report a measure of the sample size (e.g. total number or mean number of animals)
	34	Study characteristics: Report intervention/exposure details (timing, dose)
	35	Study characteristics: Report study design/intention (pharmakinetic, mechanistic, efficacy)
	36	Report the risk of bias of the primary studies (individual studies/across outcomes)
	37	Report the outcome effects of primary studies (forest plot if applicable), IF applicable^a^
	38	Report the confidence intervals of outcomes for the included studies, IF applicable^a^
	39	Report any measure of heterogeneity between studies, IF applicable^a^
	40	Report the results of sub-group and sensitivity analysis, IF applicable^a^
	41	Report the results of publication bias, OR report that it was not possible/done
**Discussion**	42	Discuss the impact of the risk of bias of the primary studies
	43	Discuss the limitations (i.e. limitation of primary studies and/or outcomes included)
	44	Discuss the limitations of the systematic review
**Other**	45	Include the funding source(s) of the systematic review
	46	Report any data sharing, OR that there was no data sharing

a Reporting item is not applicable to systematic reviews that did not perform a quantitative synthesis. For reviews that did not perform a quantitative synthesis, these items receive an NA.
